# Molecular Docking and Comparative Inhibitory Efficacy of Naturally Occurring Compounds on Vegetative Growth and Deoxynivalenol Biosynthesis in *Fusarium culmorum*

**DOI:** 10.3390/toxins13110759

**Published:** 2021-10-26

**Authors:** Safa Oufensou, Alessandro Dessì, Roberto Dallocchio, Virgilio Balmas, Emanuela Azara, Paola Carta, Quirico Migheli, Giovanna Delogu

**Affiliations:** 1 Dipartimento di Agraria, Università degli Studi di Sassari, Via E. De Nicola 9, 07100 Sassari, Italy; balmas@uniss.it (V.B.); qmigheli@uniss.it (Q.M.); 2 Nucleo di Ricerca sulla Desertificazione, Università degli Studi di Sassari, Via E. De Nicola 9, 07100 Sassari, Italy; 3 Istituto CNR di Chimica Biomolecolare, Traversa La Crucca 3, 07100 Sassari, Italy; alessandro.dessi@cnr.it (A.D.); robertonico.dallocchio@cnr.it (R.D.); emanuelagigliola.azara@cnr.it (E.A.); paola.carta@cnr.it (P.C.); giovanna.delogu@icb.cnr.it (G.D.)

**Keywords:** *Fusarium culmorum*, trichothecene B, phenols and hydroxylated biphenyls, natural compounds, plant health, food safety and security, computational studies

## Abstract

The fungal pathogen *Fusarium culmorum* causes Fusarium head blight in cereals, resulting in yield loss and contamination of the grain by type B trichothecene mycotoxins such as deoxynivalenol (DON), and its acetylated derivatives. Synthesis of trichothecenes is driven by a trichodiene synthase (TRI5) that converts farnesyl pyrophosphate (FPP) to trichodiene. In this work, 15 naturally occurring compounds that belong to the structural phenol and hydroxylated biphenyl classes were tested in vitro and in planta (durum wheat) to determine their inhibitory activity towards TRI5. In vitro analysis highlighted the fungicidal effect of these compounds when applied at 0.25 mM. Greenhouse assays showed a strong inhibitory activity of octyl gallate **5**, honokiol **13** and the combination propyl gallate **4** + thymol **7** on trichothecene biosynthesis. Docking analyses were run on the 3D model of *F. culmorum* TRI5 containing the inorganic pyrophosphate (PPi) or FPP. Significant ligand affinities with TRI-PPi and TRI-FPP were observed for the same sites for almost all compounds, with 1 and 2 as privileged sites. Octyl gallate **5** and honokiol **13** interacted almost exclusively with sites 1 and 2, by concurrently activating strong H-bonds with common sets of amino acids. These results open new perspectives for the targeted search of naturally occurring compounds that may find practical application in the eco-friendly control of FHB in wheat.

## 1. Introduction

Fusarium foot and root rot (FRR) and Fusarium head blight (FHB) are major diseases of wheat (*Triticum* L.) worldwide [1]. Several fungal species, including *Fusarium culmorum* (Wm.G. Sm.) Sacc. and *Fusarium graminearum* Schwabe, are the predominant causes of FHB and FRR. Both yield and quality losses are caused by FHB, which takes place during anthesis and develops until harvest, inducing severe contamination of the grain by mycotoxins [1,2,3,4,5].

Isolates of *F. graminearum* and *F. culmorum* may produce different type-B trichothecenes: DON (deoxynivalenol) and the acetylated forms 3- and 15-ADON (3- and 15-acetyldeoxynivalenol) or NIV (nivalenol), defining the three chemotypes reported so far [6,7,8].

Trichothecenes are chemically defined as sesquiterpene epoxides, able to cause toxicoses in humans or non-human animals that consume contaminated food or feed [9,10]. These compounds may inhibit eukaryotic protein synthesis [11], induce apoptosis [12,13] and may play a key role as virulence factors [14,15].

*TRI5* is the first biosynthetic gene ever identified in the trichothecene pathway of *F. sporotrichioides* Scherb. [16]: it encodes a sesquiterpene cyclase that catalyzes the cyclization of farnesyl diphosphate to yield hydrocarbon trichodiene (a bicyclic sesquiterpene), at least five sesquiterpene side products, and the inorganic pyrophosphate (PPi). The enzyme purified from *F. sporotrichioides* is a dimer of 45 kD subunits. Three Mg^2+^ ions are needed to activate the PPi leaving group and form a reactive allylic cation (bisabolyl carbocation), that undergoes a sudden isomerization to initiate a complex cyclization cascade through to a ligand-induced conformational change of the protein closing the entrance the active site. Binding studies suggest that the formation of the transient carbocation intermediate (farnesyl disphosphate carbocation) is under kinetic rather than thermodynamic control [17,18,19]. The X-ray crystal structure of the trichodiene synthase TRI5 complexed with Mg^2+^ (3 ions)-PPi provides useful information on the molecular recognition of PPi, hence giving more insights on the identification of external ligands hampering mycotoxin production [18,20,21].

Several fungicides (e.g., azoles and strobilurins), may control *Fusarium* spp. in the field, leading to a reduction in grain contamination, particularly when the disease pressure is low and the wheat genotype presents moderate resistance [22,23]. Nonetheless, a sharp increase in mycotoxin contamination may occur if the active ingredient is applied at low dosage or when the antifungal action towards various *Fusarium* spp. is variable [24]. Moreover, frequent application of fungicides sharing the same mechanism of action, such as in the case of sterol biosynthesis inhibitors, may exert a selective pressure on *Fusarium* populations, thereby favouring the appearance of resistant variants. Consequently, the identification of new effective molecules with different antifungal mechanisms, able to reduce both the pathogenicity and the mycotoxigenic ability of the pathogen, or to enhance host plant’s natural resistance, now represents an urgent need [25,26,27,28].

Phenolic and polyphenolic natural compounds proved effective inhibitors against trichothecene-producing strains of *Fusaria* [21,29,30,31,32,33,34,35,36,37,38,39,40,41]. Various mechanisms were proposed to explain the ability of such compounds to interfere with the biosynthesis of trichothecenes, e.g., transcriptional control of *TRI* genes [30,42], modification of the fungal membrane permeability [36,43,44], inhibition of fungal enzymes [29,45], and attenuation of oxidative stress [46].

Furthermore, phenolic compounds exert an important effect in plant defence by reinforcement of its external structure against the pathogen attack [47,48,49]. It is generally acknowledged that high content of dimers of ferulic acid in wheat contributes to durum wheat FHB resistance [49].

One of the most studied hypotheses on the role of phenolic compounds against *F. culmorum* and *F. graminearum* involves the inhibition of TRI5 [50]. The external ligand would mimic the natural substrate of the trichodiene synthase (farnesyl pyrophosphate FPP), binding to TRI5 and triggering a conformational change of the protein; this would modify or hinder its efficacy, thereby leading to a significant reduction or to the complete inhibition of trichothecene biosynthesis.

The crystal structure of TRI5 of *F. sporotrichioides* [51] was used by us to design a 3D atomic-level protein model of the *F. culmorum* TRI5 suitable to perform accurate docking studies [32]. The computational studies were performed when *F. culmorum* TRI5 contains the inorganic PPi, which is a reaction step before the entrance of the substrate FFP.

By this approach, docking data on a range of natural and natural-like phenols and hydroxylated biphenyls were integrated with in vitro [21,32,39] and field [40] assays with *F. culmorum* and *F. graminearum* to identify molecular structures, functional groups and putative amino acids most likely involved in the interaction between the phenolic molecules and the TRI5 protein. Significant inhibitory activity on trichothecene biosynthesis was reported over the 0.25–1.5 mM range of concentrations, and some of the tested molecules displayed remarkable fungicide activity [21,32,38,39,40].

Still, some differences in trichothecene inhibition or antifungal activity are observed among in silico evaluations, in vitro and field assessments. Under field conditions, we observed restoring of the trichodiene production after one week from the point application of the compound into the spikelet [40].

Aiming to better understand the structure–activity relationship between trichothecene inhibitors and TRI5 and to offer a sustainable approach to investigate new friendly inhibitors/fungicides, we have selected some phenols and hydroxylated biphenyls belonging to naturally occurring cinnamic acids, gallic esters, phenylpropanoids, monoterpenoids and phenylethanones, and evaluated in silico their binding capacity on the TRI5 protein containing the substrate (FPP) or the inorganic PPi. Further, we evaluated the in vitro and in planta activity of the compounds as fungicides or as inhibitors of trichothecene biosynthesis. Potential synergism between two natural compounds with differing mechanisms of action was also assayed. These different and complementary approaches are devoted to find a tight correlation between compounds interacting with key amino acids of TRI5 protein and their activity in inhibiting DON and 3-ADON in vitro and in planta. This common set of amino acids may play a key role in guiding the identification of TRI5 inhibitors previously proven by a proper study in silico.

## 2. Results

### 2.1. Docking of Phenols and Hydroxylated Biphenyls to TRI5

**Figure 1:**
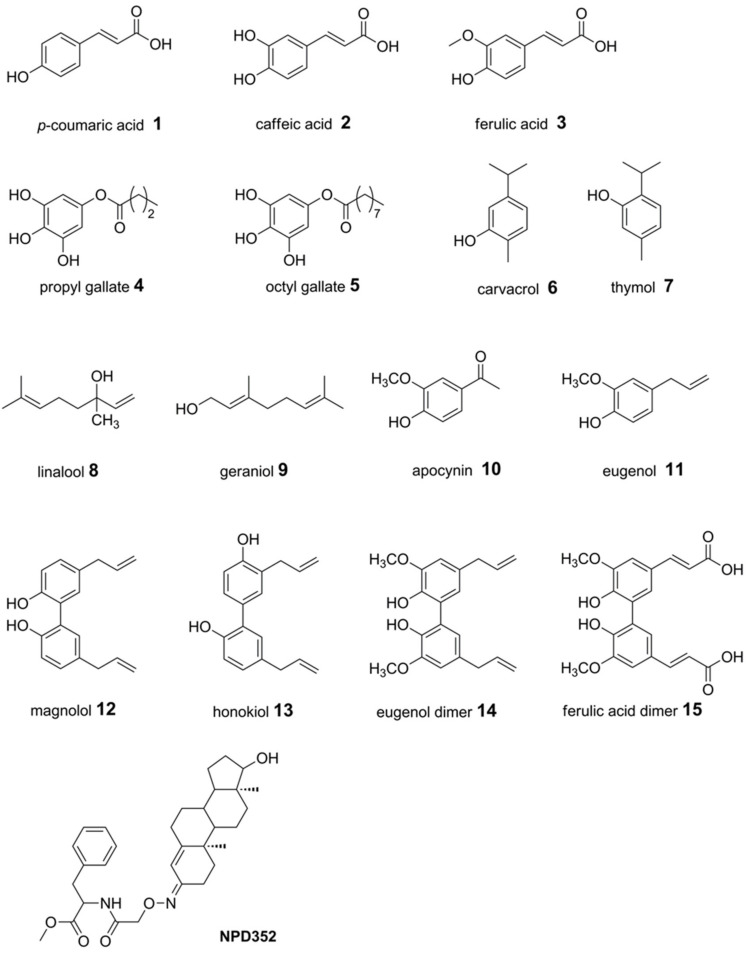
Chemical structures of compounds **1–15** and **NPD352**, a TRI5 inhibitor known in literature [52].

A set of naturally occurring compounds belonging to cinnamic acids (compounds **1**–**3** and **15**), gallic esters (**4** and **5**), terpenes (**6**–**9**), phenylpropanoids (**11**–**14**) and one phenylethanone (**10**) were selected (Figure 1). Moreover, compound **NPD352** (testosterone 3-(O-carboxymethyl)oxime amide-bonded to phenylalanine methyl ester), a trichodiene inhibitor identified by chemical array and library screening using a recombinant TRI5 expressed in *Escherichia coli* [52], was included in the docking analysis.

**Figure 2:**
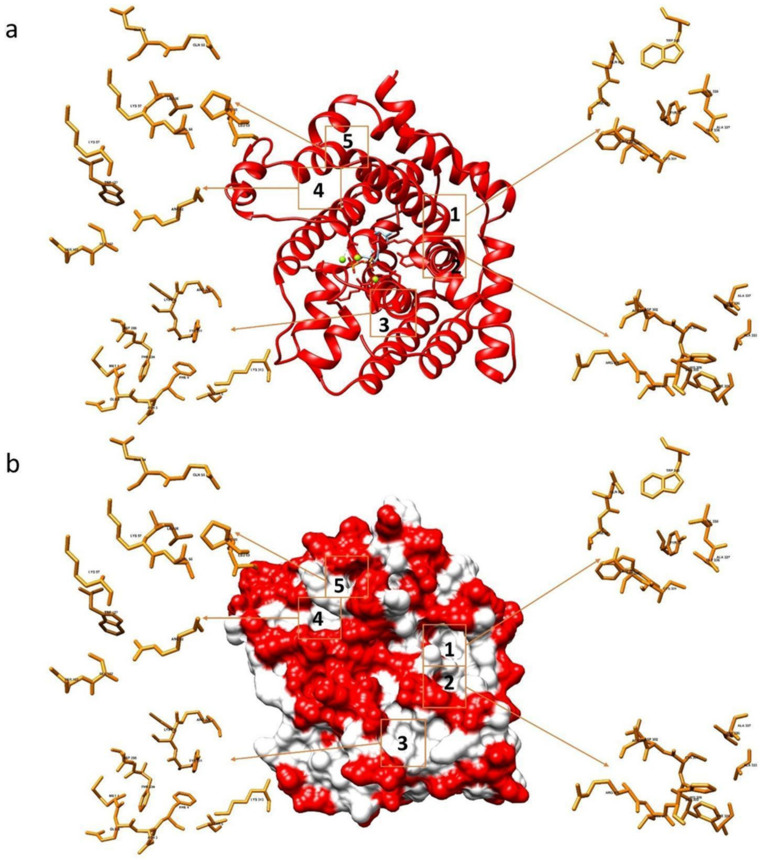
Interaction sites between the TRI5-FPP protein of the *Fusarium culmorum* model and the tested compounds. Representation in ribbons (**a**) and in surface model (**b**).

The binding capacity of compounds **1**–**15** and **NPD352** with the *F. culmorum* TRI5-PPi model [32] was investigated and the most populated sites were compared with those evaluated when the same compounds were docked with TRI5 protein containing the substrate FPP. Besides the catalytic site, five sites having significant ligand affinity were identified both on the TRI5-PPi and TRI5-FFP proteins surfaces, with preference for sites 1 and 2 (Figure 2). Glu68, Thr69, Tyr76, Cys301, Asp302, Ala337, Val338, Trp343 were amino acids assigned to site 1 and Ala303, His308, Phe329, Ala333, Gly336 to site 2 of both TRI5-PPi and TRI5-FFP.

**Figure 3:**
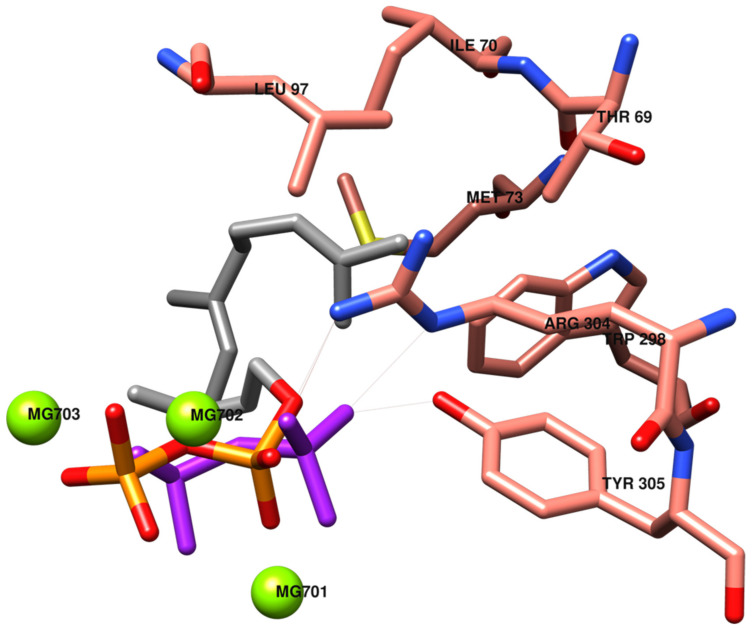
Pyrophosphate PPi (in purple) and farnesyl pyrophosphate FPP (in orange and grey) positions in the catalytic site of the TRI5 model. The most significant interactions between amino acids and the organic moiety of FPP (in grey) are evidenced.

In Figure 3, the catalytic site of TRI5 is depicted, with the inorganic moiety of substrate FPP almost overlapping on the PPi pose, activating interactions with common amino acids. The farnesyl moiety interacts with lipophilic amino acids Thr69, Ile70, Met73, Leu97, Trp298, Arg304 and Tyr305.

Differently from the TRI-PPi model, no significant binding constant (Ki) with the catalytic domain was calculated for any of the compounds **1**–**15** or **NPD352** when they interacted with the TRI-FPP model. The scoring results of the major ligand interactions with TRI5-PPi and TRI5-FPP, with similar Ki and ΔG orders of magnitude, are listed in Table 1 and Table 2, with preference for sites 1 and 2.

The ligand molecules form two main groups: (i) those characterised by a carboxyl group, likely deprotonated at physiological pH, and presenting charge -1 (compounds **1–3**) or −2 (dimer **15**); (ii) those presenting charge 0 (compounds **4–14** and **NPD352**).

The catalytic domain of TRI-PPi was preferred by cinnamic acid monomers **1** and **2**, whereas site 3 was the most populated when these compounds were docked onto TRI-FFP (Table 1 and Table 2). Ferulic acid **3** preferred sites 3 and 5 in both TRI5 configurations. On the contrary, in both TRI-PPi and TRI-FPP, ferulic acid dimer **15** simultaneously activated interactions with amino acids Glu68, Thr69, Tyr76, Cys301, Asp302, Ala337, Val338, Trp343 (site 1) and Ala303, His308, Phe329, Ala333, Gly336 (site 2) with Ki constants within the µM range (Table 1 and Table 2).

Strong H-bonds were calculated for compound **15** to Arg62, Gly68, Tyr76, Cys301, Gly336 and Val338 located in sites 1 and 2 (Tables S1 and S2). The same behaviour was observed with the inhibitor **NPD352**, that interacted almost exclusively with amino acids in sites 1 and 2 with Ki reaching a nM value (Table 1 and Table 2). Excluding Gly336 and Val338, the inhibitor **NPD352** activated almost the same H-bonds as ferulic acid dimer **15** did (Tables S1 and S2). Analogously to ferulic acid dimer **15** and ligand **NPD352**, dimers **12**–**14** preferred the set of amino acids of site 1 and 2 with which these compounds interact concurrently activating strong H-bonds with Gly336 and Val338, whereas a distinction should be made with the other set of amino acids belonging to sites 1 and 2 (Tables S1 and S2). Strong H-bonds to Trp298, Asp302, Arg306 and Leu307 were calculated only for honokiol **13** that, differently from magnolol **12**, eugenol dimer **14** and ferulic acid dimer **15**, did not activate any H-bonds with Arg62 and Tyr76, albeit binding to TyrR76 (Tables S1 and S2).

Eugenol **11** bound mainly to sites 1 and 2 but, discordantly from dimer **14**, monomer **11** interacted separately with each of the ligand-binding pockets. Apocynin **10**, structurally similar to eugenol **9** and differing for a methyl ketone group in the *para* position to the guaiacyl ring, activated strong interactions with site 1 (best docking pose) in both TRI5 configurations with similar docking score in terms of Ki and pose percentage (Table 1 and Table 2). Conversely to eugenol **11**, apocynin **10** formed H-bonds with Gln68 and Tyr76 (Table 1 and Table 2).

Although gallic esters **4** and **5** interacted with almost the same set of amino acids of sites 1 and 2 in both TRI5 configurations (Table 1 and Table 2), only octyl gallate **5** bound these two ligand-binding pockets simultaneously, activating more H-bonds than propyl gallate **4** did, especially with Tyr76, Asp302, Ala303, Leu307, Gly336 and Val338 (Tables S1 and S2).

Octyl gallate **5** activated more hydrophobic interactions with amino acids residues of sites 1 and 2 than propyl gallate **4**, magnolol **12** and thymol **7** (Figure S1, Table S3). Trp298, Asp302, Valo338, Gly336, Tyr76 and Gln68 presented high-affinity towards the four compounds. Differently from propyl gallate **4** and magnolol **12**, the main hydrophobic interactions of thymol **7** with amino acids residues concerned site 1 (Figure S1, Table S3).

Compounds **6–9** are cyclic (i.e., carvacrol **6** and thymol **7**) and acyclic (i.e., linalool **8** and geraniol **9**) monoterpenoids. Linalool **8** presents a chiral centre; therefore, docking studies were performed with both enantiomers and no significant differences were observed for *R* and *S* enantiomers in terms of ligand-binding pockets. Only in the TRI-PPi configuration did both linalool enantiomers **8** interact with the catalytic domain with docking scores ranging from 21 to 27% and Ki in a µM order (Table 1). Linalool **8** and geraniol **9** interacted with almost the same set of amino acids identified for the other compounds listed in Figure 1, except for His308 and Phe329. Differently from geraniol **9**, linalool **8** did not activate any binding with Ala333. Compounds **6**–**9** interacted mainly with amino acids in site 1, albeit with a significant docking score (Ki and pose percentage) calculated for sites 2 (carvacrol **6** and geraniol **9**) and **4** (thymol **7** and linalool **8**) (Table 1 and Table 2). No significant interactions with Glu68 located in site 1 were estimated for carvacrol **6** and thymol **7**, whereas both compounds activated an H-bond with Val338 in site 1. On the contrary, no amino acid-binding was detected in thymol **7** for Gly68, His308, Phe329, Glu330, Ala333, representative of the best ligand–amino acid interactions (Table 1 and Table 2).

### 2.2. Antifungal Activity and Trichothecene Inhibitory Effect of Tested Phenols against F. culmorum Strain UK99

#### 2.2.1. In Vitro Assay

Octyl gallate **5**, apocynin **10**, magnolol **12**, honokiol **13** and the two equimolar combination of thymol **7** with magnolol **12** and honokiol **13** at 0.25 mM completely inhibited both fungal growth and trichothecene production by *F. culmorum* UK99 (Table 3). Conversely, *p*-coumaric acid **1** and eugenol dimer **14** induced a strong production of both DON and 3-ADON (272–619% and 375–765% relative to the untreated control, respectively), with no significant effect on fungal growth. The equimolar mixture of propyl gallate **4** + thymol **7** induced a complete inhibition of trichothecene production with a fungal growth reduction of approximately 40% (Table 3). When applied alone, thymol **7** completely inhibited DON biosynthesis, as well as eugenol **11**, and both reduced the vegetative growth by 20–50%.

In the in vitro assay, detection of the pH medium was performed daily over two weeks following the addition of the compound (Table S4). After three days, a significant increase of pH in comparison to the starting liquid culture medium (Vogel’s, pH 5.8) was observed for all compounds except for propyl gallate **4**, thymol **7** and the mixture propyl gallate **4** + thymol **7**, whose medium pH decreased. In Table S4, pH and pKa of compounds **1–15** are depicted.

#### 2.2.2. Greenhouse Assay

Propyl gallate **4** applied at 4 mM allowed the highest disease index (37.76) compared to the control (63.86), followed by the equimolar solution of magnolol **12** + thymol **7** at the final concentration of 4 mM and the combination of propyl gallate **4** + thymol **7**, displaying disease indexes of 36.6 and 32.2, respectively. Thymol **7** applied alone showed a similar effect of the equimolar solution of propyl gallate **4** + thymol **7**. Magnolol **12** and honokiol **13** applied alone proved to have better efficiency, reducing the disease index by about 60% compared to the control, while octyl gallate **5** had the highest efficiency with a disease index value of 13% (Table 4).

Wheat spikes production was statistically similar for all tested compounds, including tebuconazole, for which a lower seed production compared to that observed in spikes treated with honokiol **13** at 4 mM was recorded (Table 4).

Thymol **7** reduced trichothecene contamination by approximately 30% compared to the untreated control (Table 4). The equimolar solution of magnolol **12** + thymol **7** at the final concentration of 4 mM and thymol applied alone at 4 mM reduced the DON production by 20%. When applied alone, magnolol **12** reduced DON by about 60%. Propyl gallate **4** amended alone showed a reduction of about 70%, while the equimolar combination of propyl gallate **4** + thymol **7** at a final concentration of 4 mM was the most effective treatment, leading to complete inhibition of DON biosynthesis. Octyl gallate **5** applied at 4 mM had an effect similar to that achieved by tebuconazole, and reduced DON content by 99%, followed by honokiol **13** (95%; Table 4).

## 3. Discussion

Following three complementary approaches, including in silico, in vitro and in planta assays, some naturally occurring phenols and hydroxylated biphenyls were evaluated against *F. culmorum* in order to identify common features among potential inhibitors of trichothecene biosynthesis and/or inhibitors of the fungal growth. Compounds **1–15** were selected according to the chemical structure of those tested in previous studies targeting *F. culmorum* [21,32], *F. graminearum* [40] and other *Fusarium* spp. sharing agricultural and clinical relevance [40].

Compounds **1**–**15** belong to cinnamic acids (**1**–**3** and **15**), gallic esters (**4** and **5**), monoterpenoids (**6**–**9**), phenylpropanoids (**11**–**14**) and phenylethanones (**10**). Except for **8** and **9**, all compounds share a common phenol ring with a functional group in the *para* position (i.e., compounds **1**–**5** and **10**–**15**) or in the *ortho* position (i.e., compounds **6** and **7**) to the hydroxyl group. In compounds **2**–**5** and **10**–**15**, more hydroxylated groups are present in the phenyl ring representing a catechol or guaiacyl or gallic unit, increasing the antioxidant activity and, generally, the lipophilicity. The feature of the substituent in the *para* position to the phenolic -OH group influences the lipophilicity of the compound and contributes to its ability to cross the fungal cell membrane by passive diffusion.

The antimicrobial activity of compounds **5**, **12**, **13** and, to a lesser extent, of apocynin **10**, has been established [39]. Similarly, the ability of octyl gallate **5** as a potent antimicrobial agent was demonstrated in several studies [53,54,55]. In *Aspergillus* spp., besides destabilizing cell wall integrity in the target fungi, the redox-active characteristic of octyl gallate **5** may further weaken the fungal antioxidant system [55]. Magnolol **12** and honokiol **13** display antifungal, as well as antioxidant activity, against several pathogenic fungi [39,56].

In silico, for almost all compounds, we confirmed the same binding affinity with TRI-PPi and TRI-FPP for the same sites, evidencing that 1 and 2 are privileged sites as identified previously by us in TRI-PPi [32]. A tight correlation was found for compounds interacting mainly with amino acids in sites 1 and 2 and their activity in inhibiting DON and 3-ADON in vitro and in planta.

In the in vitro assay, octyl gallate **5**, apocynin **10**, magnolol **12** and honokiol **13** totally inhibited fungal growth at 0.25 mM; nevertheless, in silico, the compounds activated interaction with a common set of amino acids of sites 1 and 2, suggesting a mycotoxin inhibitory effect. It should be considered that the binding affinity in silico is not correlated with the concentration of the ligand, therefore a compound that inhibits fungal growth at a given concentration could be a good ligand for TRI5. In this study we selected the lowest concentration previously adopted in the in vitro assay [32] because we wanted to identify compounds able to reduce, at the minimal concentration, both the pathogenicity and the mycotoxigenic ability of the pathogen without affecting the fungal growth.

Due to the chemical structure of octyl gallate **5**, magnolol **12** and honokiol **13**, these compounds interact with amino acids of sites 1 and 2, whereas apocynin **10**, structurally less flexible and dimensionally smaller, occupies site 1 almost exclusively in both TRI5 configurations. Magnolol **12** and honokiol **13** are structural isomers with a different ability to activate interactions and H-bonds with amino acids in virtue of the differing position of a phenolic -OH group, responsible for specific antioxidant activities and, in general, a different bioactivity [57]. The two phenol -OH groups present in magnolol **12** determine different acidity by forming an intramolecular H-bond interaction, making magnolol **12** less water soluble and less antioxidant than honokiol **13** [58]. As a consequence of its accentuated structural flexibility, honokiol **13** interacts almost exclusively with sites 1 and 2, whereas magnolol **12** binds also with amino acids present in sites 4 and 5. These differences between the two structural isomers were evidenced in the in planta assay, where honokiol **13** reduced the production of DON significantly more than magnolol **12**.

Based on in vitro experiments, a set of the tested compounds (i.e., ferulic acid **3**, octyl gallate **5**, carvacrol **6**, thymol **7**, apocynin **10**, eugenol **11**, magnolol **12** and honokiol **13**) inhibited trichothecene biosynthesis. These compounds activate a strong interaction with the same amino acids: Tyr76, Cys301, Asp302, Gly336, Ala337, Val338 and Trp343. **NPD352**, a TRI5 inhibitor identified by chemical array and library screening using a trichodiene synthase expressed in *Escherichia coli* as a target protein [52], interacted with the same set of amino acids as compounds **3**, **5**–**7** and **10**–**13**; this analogy offers a further proof of the reliability of our in silico TRI5 model. It is therefore reasonable to affirm that this common set of amino acids may play a key role in guiding the identification of potential non-fungicidal TRI5 inhibitors.

Compounds (**1**, **2**, **4**, **8**, **9**, **14** and **15**) that proved unable to inhibit or control trichothecene production in vitro at the tested 0.25 mM concentration deserve distinct remarks; *p*-coumaric acid **1** and caffeic acid **2** interact with ligand-binding pockets that are different from the privileged sites 1 and 2. Despite having a significant binding score, linalool **8** and geraniol **9** interact with only some of the amino acids present in sites 1 and 2. The behaviour of eugenol dimer **14** is somehow contradictory; while it interacts in silico mainly with sites 1 and 2, activating almost the same H-bonds as other strong inhibitors (e.g., octyl gallate **5**, magnolol **12** and honokiol **13**), this compound induced a six-fold increase in the in vitro production of trichothecenes. Considering the inconsistent behaviour of eugenol dimer **14** already observed in previous reports [21,32], further experiments should be targeted at evaluating the potential effects of partial solubilisation of this compound in the medium on its bioactivity.

The permeation of the phenolic compounds into the fungal membrane depends on their lipophilicity and on the pKa, i.e., the pH where the molecule is 50% deprotonated (dissociated form). Under in vitro assay conditions, except for *p*-coumaric acid **1**, caffeic acid **2** and ferulic acid **3**, all compounds exist in their undissociated form (Table S4) that, being more lipophilic than the deprotonated form, is able to cross the cell membrane by passive diffusion interfering with cellular processes [59]. The release of extracellular constituents by disruption of the fungal membrane decreases the culture medium pH, as evidenced after three days from amendment of compounds **4**, **7**, **11** and the mixture **4** + **7**, which affect fungal growth, as well as mycotoxin production. Even though the dry fungal biomass in the presence of eugenol **11** was significantly different from the control, it is likely that this compound, a weak fungicide at 0.25 mM, did not induce a drastic change in membrane permeation as compounds **4** and **7** did, maintaining a neutral pH of the liquid culture. A decrease in the pH was not observed in the presence of compounds with strong fungicidal activity that completely inhibit the fungal growth at 0.25 mM (i.e., compounds **5**, **10**, **12**, **13**, and mixtures **12** + **7** and **13** + **7**). It should be emphasized that compounds **5**, **10**, **12** and **13** are also good TRI5 inhibitors as predicted in silico and experimentally evidenced in vitro at 0.25 mM and, for octyl gallate **5** and honokiol **13**, also in planta at the concentration of 4 mM.

Propyl gallate **4**, a food antioxidant, displayed a weak fungicidal effect against *F. culmorum* in vitro, despite its reported inhibitory activity against several fungi [60]. Differently from octyl gallate **5**, propyl gallate **4** interacted in silico by activating H-bonds with the same set of amino acids identified in sites 1 and 2, albeit separately. The aliphatic chain of compound **4** is shorter compared to **5**, influencing its lipophilicity (i.e., LogP 1.51 versus 3.60, respectively), the capacity to interact with less amino acids and, ultimately, its antifungal activity [61]. Aiming to increase the inhibitory effect of propyl gallate **4**, this compound was combined in an equimolar mixture with thymol **7** at the final concentration of 0.25 mM. The antimicrobial activity of thymol is probably directed to outer and inner-membrane disruption, but also to internal sensitive comparts of the fungal cell, causing alteration in the cell membrane [62,63,64]. Furthermore, the down-regulatory effect of thymol on *TRI4* gene on *F. oxysporum* has been reported [64].

Due to the multiple action of thymol **7**, a combination of this compound with commercial fungicides was already tested, presuming that the mixture could affect different sensitive fungal targets [65,66]. We confirmed such a hypothesis by observing the complete inhibition of in vitro trichothecene biosynthesis by combining thymol **7** with propyl gallate **4**, as well as with magnolol **12** and honokiol **13**, two compounds that share a different mode of action involving oxidative stress-mediated apoptosis and mitochondrial dysfunction [67,68,69,70].

Propyl gallate **4**, octyl gallate **5**, thymol **7**, magnolol **12**, honokiol **13** and the equimolar mixtures magnolol **12** + thymol **7** and propyl gallate **4** + thymol **7** were selected for further in planta experiments. In previous field trials, the production of trichodiene—the precursor of trichothecene—was restored after one week from the point of application of the potential inhibitors on wheat spikes [40]. Based on this experimental evidence, we treated wheat spikes, artificially inoculated with *F*. *culmorum* spores, with the potential inhibitor every week for three times consecutively. Aiming to increase the inhibitory effect of the compounds without affecting the crop, we chose a higher concentration (4 mM) in comparison to that selected previously [40]. Complete inhibition of trichothecene in wheat grain and low severity in comparison to the control was observed upon treatment with octyl gallate **5**, honokiol **13** and propyl gallate **4** + thymol **7**.

In order to improve water solubility of the phenolic molecules and their bioavailability in planta, an aqueous mixture of β-cyclodextrin (β-CD) and phytic acid, at a non-phytotoxic concentration, was used as formulating agent. Due to a truncated conical shape with a hydrophobic cavity and an external hydrophilic surface, β-CD can interact with lipophilic molecules, facilitating their delivery in aqueous solutions. Due to these properties and its relative non-toxicity, β-CDs have been largely used in medicine, food and materials. The mixture of β-CD and phytic acid did not affect fungal growth in vitro (data not shown).

The different behaviour of magnolol **12** in vitro and in planta assays is worthy of some consideration. In vitro, the fungus is exposed to an unfavourable environment because it grows in close contact with the potential inhibitor, both diluted in the same medium; on the contrary, in planta, the effect of the compound is weaker, due to the need to reach the fungus within the plant tissue. Lipophilicity, antioxidant activity of the compound, and composition of the carrier solution are key elements to magnify the effect of the potential inhibitor in planta and previously identified by in silico evaluation. In our in planta experiments, octyl gallate **5** and the mixture propyl gallate **4** + thymol **7** satisfies all these requirements. The compounds are generally recognized as safe (GRAS list) and commercially available at low price, therefore they represent promising agents to be applied as DON inhibitors in wheat.

## 4. Conclusions

To summarize, in this work, 15 naturally occurring compounds were evaluated in silico with TRI5-PPi and TRI5-FPP models in order to identify the amino acid clusters responsible for inhibiting trichothecene production by *F. culmorum*. Nine of them determined complete inhibition of the fungal target in vitro at 0.25 mM, and a correlation was evidenced with data carried out in silico identifying a set of amino acids, namely Tyr76, Cys301, Asp302, Gly336, Ala337, Val338 and Trp343, which play a key role in the production of trichothecene. The compounds interacted with these amino acids residues with a binding constant that ranges from approximately 5 to 200 µM. Equimolar mixtures of thymol **7**, a known antimicrobial agent with multiple modes of action, and some of the tested compounds were assayed in vitro and in planta. Octyl gallate **5**, honokiol **13** and the equimolar mixture propyl gallate **4** + thymol **7** proved effective in planta by inhibiting trichothecenes up to 95–99% and in increasing wheat seed production up to 25–40% without any significant phytotoxic effect. Propyl gallate **4**, octyl gallate **5** and thymol **7** are widely used as food additives and are commercially available at a reasonable price; our results offer new insights into the search for naturally occurring compounds that may find practical application in the eco-friendly control of FHB in wheat.

## 5. Materials and Methods

### 5.1. Fungal Strain and Culture Conditions

The wild-type strain FcUK99 of *F. culmorum* (Rothamsted Research, Harpenden, UK, NRRL54111) was used in in vitro and in glasshouse experiments. This strain produces mainly 3-ADON and lower amounts of deoxynivalenol [71]. The strain was maintained as described previously [21].

### 5.2. Selected Phenolic Compounds

Compounds **1**–**15** (Figure 1) were used with purity >98% and tested at the concentration of 0.25 mM in vitro and at 4 mM in greenhouse experiments. In in vitro assay, linalool **8** was used in racemic form, whereas both enantiomers R and S were evaluated in docking studies.

Compounds **1**–**11** were purchased by Sigma-Aldrich (Milan, Italy), whereas magnolol **12** and honokiol **13** were purchased by Chemos GmbH, Merck (Regenstauf, Germany). Eugenol dimer **14** and ferulic acid dimer **15** were prepared with light modifications, according to [72,73] respectively. β-CD (CAVAMAX^®^7 PHARMA; Wacker Chemie Italia, Peschiera Borromeo, Italy) was amended in the medium to improve solubility of phenolic molecules.

### 5.3. Amendment with Compounds **1**–**15** and Sample Preparation for DON Analysis

The experiments were carried out as described by [21], with some modification as follows: Petri plates (S.I.A.L, Rome, Italy) (90 Ø) containing 10 mL of Vogel’s medium [74] were amended with each of the compounds at a final concentration of 0.25 mM. Previously, the compound was dissolved in a 6 mM aqueous solution of β-CD and 5 mL of the solution added to Vogel’s medium, reaching 10 mL of final solution. The procedure was performed in a way to exclude any stable inclusion of the compound into the βCD cavity. Each sample vial was sonicated at room temperature (RT) for 60 min and 60 Hz (Branson model 3510 OPTO-LAB, Modena, Italy). Sonicated media were inoculated with a FcUK99 spore suspension to achieve a final concentration of 1 × 10^4^ CFU/mL. Each compound was tested in five replicates, and cultures were incubated in the dark at 25 °C, without shaking. The evolution of pH in Vogel’s medium amended with the different compounds was recorded at 0, 1, 3, 4, 5, 6, 8, 10 and 14 days (d) (Table S4).

### 5.4. Greenhouse Assay

A greenhouse assay was conducted on some compounds applied alone (propyl gallate **4**, octyl gallate **5**, thymol **7**, magnolol **12**, honokiol **13**) or in combination (propyl gallate **4** + thymol **7**, magnolol **12** + thymol **7**) at the Centre of Conservation and Valorisation of Plant Biodiversity (Alghero, Italy), University of Sassari. The *Triticum durum* cv. “Saragolla”, susceptible to *F. culmorum*, was used. Six wheat spikes were sprayed with selected compounds at 4 mM. β-CD and phytic acid (Sigma-Aldrich, Darmstadt, Germany) were added as formulating agents to the compounds at 1.5 μg/mL and 8 μg/mL, respectively, to improve water solubility of the phenolic molecules. A treatment with tebuconazole (4 mM a.i.) was considered in order to compare the efficiency of the natural compounds.

A total of 2 mL was dispensed on each spike. Treated spikes were spray-inoculated with 250 μL of 10 ^4^ CFU/mL of FcUK99 inoculum 24 h later, and subsequently covered by plastic bags for 48 h in order to create adequate moisture for infection. Four replicates were performed for each compound and the treatment has been repeated three times, with an interval of one week. In this experiment, disease index (McKinney index), grain production (g) and total DON (including DON and the acetylated form 3-ADON) production were evaluated. Trichothecenes were quantified by LC-MS analysis.

### 5.5. Analytical Method, Instruments and Equipment for The Detection of Type B Trichothecenes

#### 5.5.1. Sample Preparation

Wheat seeds (5.0 g) were finely ground and transferred into a 50 mL centrifuge tube with 10 mL of water; 10 mL of 10% (*v*/*v*) HOAc in ACN was added and vortexed for 1 min at high speed. Phenomenex extraction kit KSO-8909 (Agilent Technologies, Santa Clara, CA, USA) (4.0 g MgSO_4_, 1.0 g NaCl, 1.0 g sodium citrate tribasic dehydrate SCTD and 0.5 g sodium citrate dibasic sesquihydrate SCDS) was added and hand-shaken for 1 min. The resulting mixture was centrifuged at 4000 rpm for 5 min, and the supernatant transferred into a 15 mL centrifuge tube containing KSO-8924 purification kit (900 mg MgSO_4_, 150 mg PSA). The mixture was then shaken over a vortex mixer (3000 rpm for 30 s) and centrifuged at 4000 rpm for 5 min. An aliquot (2 mL) of the supernatant was evaporated to dryness under a nitrogen gas stream and reconstituted in 1 mL of LC-MS mobile phase (A: 5 mM ammonium acetate with 0.5% acetic acid, B: 5 mM ammonium acetate in methanol with 0.5% acetic acid). The extracts were filtered through 0.22 µm PTFE syringe filters prior to LC-MS analysis.

#### 5.5.2. Q-Orbitrap-HRMS Analyses

Chromatographic separation of mycotoxins was performed on Agilent 1200 LC (Agilent Technologies, Santa Clara, CA, USA). The mobile phase consisted of: Eluent A: 5 mM ammonium acetate, 0.5% acetic acid; and B: 5 mM ammonium acetate in methanol, 0.5% acetic acid. The gradient elution was performed at the flow rate of 0.4 mL min^−1^ and a run time of 11 min. Time program = 0 min, 95% A/B; 2 min, 95% A/B; 5 min, 70% A/B; 10 min, 70% A/B. Injection volume: 5 µL. The column was then washed for 3 min with 50% B, reconditioned for 3 min by using the initial composition of mobile phases. The column compartment was maintained at 37 °C.

The HPLC system was coupled to a Q Exactive™ Hybrid Quadrupole-Orbitrap High Resolution Mass Spectrometer HRMS (Thermo Fisher Scientific, San Jose, CA, USA). HRMS was equipped with a heated electrospray ionization source (HESI-II) and operated in the full scan (*m*/*z* 100–600) with a resolving power of 140,000 FWHM. The HESI parameters conditions were: spray voltage, 3.3 kV; sheath gas flow rate (N2) 35 units; capillary temperature 300 °C; S-lens RF level 50; heater temperature 350 °C. The automatic gain control (AGC) was set at 1 × 10^−6^, and the maximum inject time was 0.1 s. Nitrogen (nitrogen generator Zefiro; Clantecno-logica, Seville, Spain) was used as both collision and damping gas.

External calibration was achieved by Pierce™ negative/positive ion calibration solution (Thermo Fisher Scientific, Rockford, IL, USA) every three days to keep a working mass accuracy lower than/equal to 5 ppm. XCalibur 2.2 and Trace Finder 3.0 (Thermo Fisher Scientific, San Jose, CA, USA) were used for LC-MS control and data processing, respectively.

The limit of detection (LOD) was determined by injecting scalar dilution of analyte standards in triplicate and analysing the standard deviation of the response and slope. Lower limit of detection was calculated as: LOD = 3.3 σ/S, where σ = standard deviation of the response, calculated as the standard deviation of the y-intercepts of the regression line, and S = slope of the calibration curve. The LOQ is the lowest assessed concentration which can reproducibly give an analyte response that is both accurate (100 ± 20% recovery) and precise (≤20% Relative Standard Deviation RSD). The LOD and LOQ values for tricothecenes were reported respectively as shown in Table 5.

### 5.6. Statistical Analysis

The results obtained from separate experiments were represented as the mean and standard error (SE) of at least four replicated measurements. The significant differences between treatments were statistically evaluated by SE and one-way analysis of variance (ANOVA) using Minitab (Minitab Ltd., Brandon Court, UK) for Windows, release 17. The data between two different treatments were compared statistically by ANOVA, followed by Dunnett test (Table 3) or Tukey test (Table 4) if the ANOVA result was significant at *p* < 0.05.

### 5.7. Molecular Docking: In Silico Analysis of Selected Phenols-TRI5 Binding

A model of *F. culmorum* TRI5 was previously developed [32] based on homology modelling with the crystallized protein of *F. sporotrichioides* (PDB code 1JFG, https://www.rcsb.org/structure/1JFG, deposited: 20 June 2021 [51]) and the starting TRI5 protein sequence of *F. culmorum* (UniProtKB/Swiss-Prot databases, accession number Q8NIG9, https://www.uniprot.org/uniprot/Q8NIG9, Sequence version: 10 January 2002). Computational modelling was performed on an HP8100 Workstation (Hewlett-Packard Company 3000, Palo Alto, CA, USA) and EXXACT Tensor Workstation (EXXACT Corporation, Fremont, CA, USA) TWS-1686525-AMB with the Cuda platform (Cuda form NVIDIA Corporation, Santa Clara, CA, USA) with OS Ubuntu 18.04, 20.04(Canonical Corporation, London, UK) CentOS 6 (CentOS Project, Red Hat, Inc, Raleigh, NC, USA) or Windows 10 (Microsoft Corporation, Washington, DC, USA). The complete methodology has already been described by [32]. Hydrophobic interactions were obtained using LigPlot + software (European Bioinformatics Institute, Cambridge, UK; https://www.ebi.ac.uk/thornton-srv/software/LigPlus/, accessed on 21 October 2021) [75].

To allow the evaluation of a large number of interactions in ligands with different docking sites, docking was run by considering a wide grid centered in the catalytic site, including almost the entire *F. culmorum* TRI5 enzyme. A 0.375 Å grid spacing was adopted, treating the docking active site as a rigid system and the ligands as flexible with free rotation around single bonds.

The reliability of the docking approach was verified by extracting the organic substrate (FPP) from the catalytic site and by considering it as a normal ligand, similarly to the TRI-PPi model [32]. In TRI-FPP docking calculations, FPP was considered as an additional fixed residue. The TRI-FPP model was built up by docking FPP, conformationally and energetically optimized, with TRI5 lacking the PPi and considering the pose of FPP with 59% of probability. A further proof of the reliability of the in silico TRI5 model was obtained by docking compound **NPD352**, a TRI5 inhibitor identified by chemical array and library screening using a trichodiene synthase expressed in *Escherichia coli*, as a target protein [52]. **NPD352** interacted with the same set of amino acids as compounds **3**, **5**–**7** and **10**–**13**.

### 5.8. Estimation of Selected Physicochemical Descriptors

Lipophilicity of compounds **1**–**19** was estimated by ChemBioDraw Ultra (Perkin Elmer Informatics, Every, France) 13.0 software using the logarithm of the partition coefficient for n-octanol/water (LogP) and listed in Table S1.

## Figures and Tables

**Table 1:** Docking for TRI5 containing the inorganic pyrophosphate (PPi).

PPi					
Tested Ligands	%	Sites	E.F.E.B. ^a^	E.I.C., Ki ^b^	Interaction with Amino Acids
*p*-Coumaric acid **1**	75	c.d. ^c^	−13.23	199.19 pM	Asp100 Glu164 Pro178 Leu181 Arg182 Asn185 Asp239 Ile241 Ser242 Leu243 Asn246 PPi700 Mg702 Mg703
	1	c.d.	−7.73	2.17 uM	Ile70 Met73 Tyr93 Leu97 Asp100 Phe157 Arg182 Asn185 Leu187 PPi700 Mg703
	4	3	−5.59	79.22 uM	Met1 Glu2 Asn3 Phe4 Thr6 Phe234 Asp235 Lys313
	11	3	−5.56	84.50 uM	Phe4 Thr6 Tyr231 Phe234 Asp235 Arg306 Lys313
Caffeic acid **2**	39	c.d.	−12.06	1.45 nM	Asp100 Glu164 Leu181 Arg182 Asn185 Asp239 Ile241 Ser242 Leu243 Asn246 PPi700 Mg702 Mg703
	16	4	−5.81	55.08 uM	Met55 Leu56 Lys57 Arg62 Val98 Ser102 Ser103 Pro126 Trp127
	11	3	−5.45	101.75 uM	Met1 Glu2 Asn3 Phe4 Thr6 Thr231 Phe234 Asp235 Lys313
Ferulic acid **3** [32]	2	c.d.	−9.62	89.12 nM	Asp100 Glu164 Leu181 Arg182 Asn185 Asp226 Glu233 Arg238 Asp239 Ser242 Leu243 Asn246 PPi700 Mg703
	6	c.d.	−7.78	1.99 uM	Ile70 Met73 Tyr93 Thr96 Asp100 Phe157 Arg182 Asn185 Leu187 PPi700 Mg703
	13	3	−5.58	80.65 uM	Met1 Glu2 Asn3 Phe4 Tyr231 Lys232 Phe234 Asp235 Arg306
	8	4	−5.51	92.16 uM	Met55 Leu56 Lys57 Arg62 Val98 Ser102 Ser103 Pro126 Trp127
	14	3	−5.48	96.45 uM	Phe4 Thr6 Tyr231 Phe234 Asp235 Arg306 Lys313
	19	5	−5.19	157.79 uM	Gln53 Gln54 Leu56 Lys57 Val58 Pro60 Leu63
Propyl gallate **4** [32]	40	1	−5.86	50.71 uM	Ala65 Gln68 Thr69 Tyr76 Trp298 Cys301 Asp302 Ala303 Gly336 Val338 Pro340 Trp343
	13	2	−5.28	133.84 uM	Leu300 Cys301 Asp302 Ala303 Arg306 Leu307 His308 Phe329 Ala333 Ala337
Octyl gallate **5**	12	2-1	−6.42	19.85 uM	Gly72 Tyr76 Leu300 Cys301 Asp302 Ala303 Arg306 Leu307 His308 Phe329 Gly336 Ala337 Val338
	9	1	−6.30	23.91 uM	Ala65 Ser66 Gln68 Thr69 Tyr76 Cys301 Asp302 Ala303 Arg304 Gly336 Val338 Trp343
	16	1-2	−6.28	24.94 uM	Gln68 Thr69 Gly72 Cys301 Asp302 Ala303 Leu307 His308 Phe329 Gly336 Ala337 Val338 Trp343
	5	1-2	−5.83	53.08 uM	Leu36 Gly72 Tyr76 Leu300 Cys301 Asp302 Ala303 Arg306 Leu307 His308 Gly336 Val338 Trp343
Carvacrol **6**	48	1	−5.31	127.65 uM	Leu36 Gln68 Thr69 Gly72 Tyr76 Cys301 Asp302 Gly336 Ala337 Val338 Trp343
	21	2	−5.29	132.10 uM	Leu300 Cys301 Asp302 Ala303 Arg306 Leu307 His308 Phe329 Ala333
	11	c.d.	−5.27	136.69 uM	Met73 Tyr93 Thr96 Leu97 Asp100 Phe157 Leu187 Val191 Met221
	16	4	−5.18	160.32 uM	Met55 Lys57 Arg62 Val98 Leu99 Ser102 Asp104 Pro126 Trp127
Thymol **7**	5	c.d.	−5.63	74.28 uM	Glu164 Pro178 Leu181 Arg182 Asn185 Asp226 Glu233 Ile241 Ser242 Leu243 Asn246 PPi700
	20	4	−5.50	92.72 uM	Met55 Leu56 Lys57 Val58 Val98 Leu99 Asp104 His125 Pro126 Trp127
	58	1	−5.34	121.84 uM	Tyr76 Trp298 Cys301 Asp302 Gly336 Val338 Pro340 Trp343
	10	c.d.	−5.17	161.91 uM	Ile70 Met73 Tyr93 Thr96 Leu97 Asp100 Phe157 Leu187 Val191 PPi700
*R*-Linalool **8**	36	1	−5.32	126.02 uM	Leu36 Gln68 Thr69 Gly72 Tyr76 Cys301 Asp302 Gly336 Ala337 Val338 Trp343
	21	c.d.	−5.31	128.71 uM	Ile70 Met73 Trp78 Tyr93 Thr96 Leu97 Phe157 Leu187 Val191 Met221 Tyr295
	12	1	−5.24	145.29 uM	Leu36 Ala65 Gln68 Thr69 Gly72 Tyr76 Asp302 Ala303 Trp343
	10	4	−5.07	193.14 uM	Met55 Leu56 Lys57 Val98 His125 Pro126 Trp127
*S*-Linalool **8**	11	4	−5.26	140.56 uM	Met55 Leu56 Lys57 Arg62 Val98 His125 Pro126 Trp127
	36	1	−5.22	148.58 uM	Gln68 Thr69 Tyr76 Cys301 Asp302 Gly336 Val338 Pro340 Trp343
	27	c.d.	−5.18	160.63 uM	Ile70 Met73 Trp78 Tyr93 Thr96 Leu97 Val191 Met221 Tyr295
	17	1	−5.14	171.53 uM	Ala65 Gln68 Thr69 Gly72 Tyr76 Cys301 Asp302 Ala303 Trp343
Geraniol **9**	2	c.d.	−6.60	14.53 uM	Pro178 Leu181 Arg182 Asn185 Glu233 Arg238 Asp239 Ile241 Ser242 Leu243 Asn246 PPi700 Mg702 MGg703
	12	2	−5.42	106.54 uM	Leu300 Cys301 Asp302 Ala303 Tyr305 Arg306 Leu307 His308 Glu309 Phe329
	47	1	−5.30	131.30 uM	Gln68 Thr69 Gly72 Tyr76 Cys301 Asp302 Gly336 Ala337 Val338 Pro340 Trp343
	16	c.d.	−5.04	200.93 uM	Ile70 Met73 Tyr93 Thr96 Leu97 Phe157 Arg182 Leu187 Met221 Asn225 Tyr295 Trp298 Tyr305 PPi700
Apocynin **10** [32]	4	c.d.	−6.86	9.29 uM	Asp100 Glu164 Arg182 Asn185 Asp226 Glu233 Arg238 Asp239 Ser242 Leu243 PPi700 Mg703
	60	1	−5.35	119.64 uM	Gln68 Tyr76 Cys301 Asp302 Gly336 Ala337 Val338 Trp343
	11	1	−5.25	134.28 uM	Gln68 Tyr69 Gly72 Tyr76 Trp298 Cys301 Asp302 Gly336 Ala337 Val338
Eugenol **11** [32]	3	c.d.	−5.65	72.36 uM	Asp100 Glu164 Leu181 Arg182 Asn185 Asp226 Glu233 Arg238 Asp239 Ile241 Ser242 Leu243 Asn246 PPi700
	31	2	−5.12	177.48 uM	His299 Leu300 Cys301 Asp302 Ala303 Arg306 Leu307 His308 Glu309 Phe329
	19	2	−5.07	193.10 uM	His299 Leu300 Cys301 Asp302 Ala303 Arg306 Leu307 His308 Phe329 Ala333 Ala337
	22	1	−4.98	224.72 uM	Gln68 Thr69 Tyr76 Trp298 Cys301 Asp302 Gly336 Ala337 Val338 Trp343
Magnolol **12** [32]	29	1	−6.91	8.56 uM	Gln68 Thr69 Gly72 Tyr76 Leu300 Cys301 Asp302 Phe329 Gly336 Ala337 Val338 Pro340
	11	1-2	−6.49	17.43 uM	Leu36 Gly72 Tyr76 Trp298 Leu300 Cys301 Asp302 Ala303 Phe329 Ala333 Ala337 Val338 Pro340 Trp343
	14	4	−5.91	46.77 uM	Met55 Leu56 Lys57 Val58 Arg62 Val98 Ser102 Ser103 Pro126 Trp127
Honokiol **13**	25	1-2	−7.25	4.81 uM	Gln68 Thr69 Gly72 Trp298 Leu300 Cys301 Asp302 Phe329 Ala333 Ala337 Val338 Trp343
	6	2	−7.15	5.72 uM	Leu300 Cys301 Asp302 Ala303 Tyr305 Arg306 Leu307 His308 Tyr311 Phe329 Glu330 Ala333 Ala337
	24	1-2	−7.04	6.91 uM	Ala65 Gln68 Thr69 Gly72 Tyr76:Trp298 Cys301 Asp302 Ala303 Gly336 Ala337 Val338 Pro340
	16	1-2	−6.92	8.44 uM	Gln68 Thr69 Gly72 Tyr76 Trp298 Cys301 Asp302 His308 Ala337 Val338
Eugenol dimer **14** [32]	14	1-2	−6.69	12.41 uM	Gln68 Gly72 Tyr76 Trp298 Cys301 Asp302 Ala303 His308 Ala337 Val338
	28	1-2	−6.67	12.98 uM	Thr69 Gly72 Tyr76 Trp298 Leu300 Cys301 Asp302 Ala303 Phe329 Ala333 Ala337 Val338 Ala339 Pro340 Trp343
	10	1-2	−6.27	25.41 uM	Leu36 Gln68 Thr69 Tyr76 Leu300 Cys301 Asp3Ala303 Phe329 Ala333 Gly336 Ala337 Val338 Trp343
	14	4	−5.41	108.76 uM	Lys57 Val58 Arg62 Val98 Leu99 Ser102 Ser103 Asp104 Pro126 Trp127
Ferulic acid dimer **15**	4	1-2	−6.48	17.71 uM	Leu36 Ala65 Gln68 Thr69 Gly72 Tyr76 Cys301 Asp302 Ala303 Arg304 Ala337 Pro340 Trp343
	26	2-1	−5.55	85.48 uM	Gln68 Thr69 Tyr76 Trp298 Cys301 Asp302 Ala303 Gly336 Ala337 Val338 Ala339 Pro340
NPD352	6	1-2	−9.52	104.59 nM	Tyr76 Cys301 Asp302 Ala303 His308 Tyr311 Phe329 Glu330 Ala333 Ala337 Val338 Pro340 Trp343
	22	1-2	−8.05	1.25 uM	Gly72 Tyr76 Cys301 Asp302 Ala303 His308 Phe329 Ala333 Ala337 Val338 Ala339 Trp343

^a^ E.F.E.B.: Estimated Free Energy of Binding, ^b^ E.I.C., Ki: Estimated Inhibition Constant, Ki., ^c^ c.d.: catalytic domain.

**Table 2:** Docking for TRI5 containing the farnesyl pyrophosphate (FPP).

FPP					
Tested Ligands	%	Sites	E.F.E.B. ^a^	E.I.C., Ki ^b^	Interaction with Amino Acids
*p*-Coumaric acid **1**	27	3	−5.50	92.97 uM	Glu2 Phe4 Thr6 Phe234 Asp235 Lys313
	45	3	−5.47	98.12 uM	Phe4 Thr6 Phe234 Asp235 Arg306 Lys313
	22	5	−5.21	139.08 uM	Gln54 Leu56 Lys57 Val58 Pro60 Leu63
Caffeic acid **2**	20	4	−5.62	75.82 uM	Met55 Leu56 Lys57 Arg62 Val98 Ser103 Pro126 Trp127
	15	5	−5.48	96.25 uM	Gln54 Leu56 Lys57 Val58 Pro60 Leu63
	25	3	−5.35	119.73 uM	Met1 Glu2 Asn3 Phe4 Thr6 Tyr231 Phe234 Asp235 Lys313
Ferulic acid **3**	16	3	−5.72	64.31 uM	Met1 Glu2 Phe4 Thr6 Tyr231 Phe234 Asp235 Lys313
	9	4	−5.38	113.66 uM	Met55 Leu56 Lys57 Arg62 Val98 Ser102 Ser103 Pro126 Trp127
	23	5	−5.14	170.90 uM	Gln53 Gln54 Leu56 Lys57 Val58 Pro60 Leu63
	8	3	−5.13	173.60 uM	Met1 Glu2 Asn3 Phe4 Tyr231 Lys232 Phe234 Asp235 Arg306
	19	3	−4.78	313.10 uM	Phe4 Thr6 Tyr231 Phe234 Asp235 Arg306 Lys313
	9	2	−4.51	491.13 uM	Leu300 Cys301 Asp302 Ala303 Tyr305 Arg306 Leu307 His308 Phe329 Ala333 Ala337
Propyl gallate **4**	2	1	−5.72	64.62 uM	Gln68 Thr69 Gly72 Tyr76 Cys301 Asp302 Gly336 Val338 Pro340 Trp343
	39	1	−5.55	85.47 uM	Ala65 Gln68 Thr69 Tyr76 Cys301 Asp302 Ala303 Gly336 Val338 Trp343
	16	2	−5.29	131.61 uM	Cys301 Asp302 Ala303 Arg306 Leu307 His308 Phe329 Ala333 Ala337
	7	1-2	−5.04	201.42 uM	Gly72 Tyr76 Cys301 Asp302 Ala333 Gly336 Ala337 Val338
	12	4	−4.36	632.98 uM	Lys57 Val58 Asp59 Arg62 Ser103
Octyl gallate **5**	12	2-1	−6.53	16.47 uM	Tyr76 Leu300 Cys301 Asp302 Ala303 Tyr305 Arg306 Leu307 His308 Phe329 Ala333 Gly336 Ala337 Val338
	15	1-2	−6.25	26.31 uM	Gly72 Tyr76 Leu300 Cys301 Asp302 Arg306 Leu307 His308 Phe329 Gly336 Ala337 Val338
	10	1	−6.24	26.46 uM	Ala65 Ser66 Gln68 Thr69 Tyr76 Cys301 Asp302 Ala303 Arg304 Gly336 Val338 Trp343
	9	2-1	−5.65	72.30 uM	Tyr76 Leu300 Cys301 Asp302 Ala303 Tyr305 Arg306 Leu307 His308 Phe329
Carvacrol **6**	62	1	−5.31	127.77 uM	Leu36 Gln68 Thr69 Gly72 Tyr76 Asp302 Gly336 Ala337 Val338 Trp343
	24	2	−5.29	132.22 uM	Cys301 Asp302 Ala303 Arg306 Leu307 His308 Phe329 Ala333
	9	4	−5.18	158.87 uM	Met55 Lys57 Arg62 Val98 Leu99 Asp104 Pro126 Trp127
Thymol **7**	18	4	−5.51	91.27 uM	Met55 Leu56 Lys57 Val58 Val98 Leu99 Asp104 His125 Pro126 Trp127
	72	1	−5.34	120.99 uM	Tyr76 Trp298 Cys301 Asp302 Gly336 Val338 Pro340 Trp343
	6	1	−5.14	171.69 uM	Leu36 Gly72 Tyr76 Cys301 Asp302 Ala303 Gly336 Val338 Trp343
R-Linalool **8**	11	1	−5.22	148.63 uM	Leu36 Ala65 Gln68 Thr69 Gly72 Tyr76 Asp302 Ala303 Trp343
	60	1	−5.13	172.51 uM	Gln68 Thr69 Tyr76 Gly336 Ala337 Val338 Pro340 Trp343
	9	4	−5.06	196.45 uM	Met55 Leu56 Lys57 Val98 His125 Pro126 Trp127
	9	1	−4.92	248.45 uM	Leu36 Gly72 Tyr76 Cys301 Asp302 Gly336 Ala337 Val338 Trp343
S-Linalool **8**	8	4	−5.30	131.37 uM	Met55 Leu56 Lys57 Arg62 Val98 His125 Pro126 Trp127
	55	1	−5.25	142.97 uM	Gln68 Thr69 Tyr76 Cys301 Asp302 Gly336 Ala337 Val338 Pro340 Trp343
	25	1	−5.22	148.04 uM	Leu36 Ala65 Gln68 Thr69 Gly72 Tyr76 Cys301 Asp302 Ala303 Trp343
Geraniol **9**	13	2	−5.40	110.42 uM	Leu300 Cys301 Asp302 Ala303 Tyr305 Arg306 Leu307 His308 Glu309 Phe329
	55	1	−5.31	128.06 uM	Gln68 Thr69 Gly72 Tyr76 Cys301 Asp302 Gly336 Ala337 Val338 Pro340 Trp343
	7	1	−4.99	218.54 uM	Gln68 Gly72 Tyr76 Cys301 Asp302 Ala303 Gly336 Trp343
	12	4	−4.98	225.19 uM	Met55 Leu56 Lys57 Arg62 Val98 Ser103 His125 Pro126 Trp127
Apocynin **10**	67	1	−5.39	111.78 uM	Gln68 Tyr76 Asp302 Gly336 Ala337 Val338 Trp343
	12	1	−5.32	125.59 uM	Gln68 Thr69 Gly72 Tyr76 Cys301 Asp302 Gly336 Ala337 Val338
	11	4	−5.15	168.53 uM	Met55 Leu56 Lys57 Arg62 Val98 Ser103 Asp104 Trp127
Eugenol **11**	27	2	−5.20	153.84 uM	His299 Leu300 Cys301 Asp302 Ala303 Tyr305 Arg306 Leu307 His308 Phe329 Ala333
	23	2	−5.16	165.71 uM	His299 Leu300 Cys301 Asp302 Ala303 Arg306 Leu307 His308 Glu309 Phe329
	32	1	−5.02	208.24 uM	Gln68 Thr69 Tyr76 Trp298 Cys301 Asp302 Gly336 Ala337 Val338 Trp343
	12	4	−4.81	296.34 uM	Met55 Leu56 Lys57 Arg62 Val98 Ser102 Asp104 His125 Pro126 Trp127
Magnolol **12**	38	1-2	−6.93	8.36 uM	Gln68 Thr69 Tyr76 Leu300 Cys301 Asp302 Ala303 Phe329 Ala333 Gly336 Ala337 Val338 Pro340 Trp343
	14	1-2	−6.79	10.59 uM	Gln68 Thr69 Gly72 Tyr76 Cys301 Asp302 Ala303 Gly336 Ala337 Val338 Trp343
	10	1-2	−6.46	18.40 uM	Gly72 Tyr76 Trp298 Leu300 Cys301 Asp302 His308 Phe329 Gly336 Ala337 Val338 Pro340
	17	4	−5.87	50.12 uM	Met55 Leu56 Lys57 Arg62 Val98 Ser102 Ser103 His125 Pro126 Trp127
Honokiol **13**	23	1-2	−7.25	4.89 uM	Gln68 Thr69 Gly72 Trp298 Leu300 Cys301 Asp302 Phe329 Ala337 Val338 Pro340 Trp343
	13	2	−7.14	5.87 uM	Leu300 Cys301 Asp302 Ala303 Tyr305 Arg306 Leu307 His308 Tyr311 Phe329 Glu330 Ala333 Ala337
	23	1	−7.01	7.31 uM	Leu36 Ala65 Gln68 Thr69 Gly72 Tyr76 Cys301 Asp302 Ala303 Gly336 Ala337 Val338 Trp343
	15	1-2	−6.93	8.36 uM	Gln68 Thr69 Gly72 Tyr76 Trp298 Cys301 Asp302 His308 Phe329 Ala337 Val338
Eugenol dimer **14**	35	1-2	−6.99	7.58 uM	Gln68 Gly72 Tyr76 Trp298 Cys301 Asp302 Ala303 His308 Ala337 Val338 Trp343
	19	1-2	−6.85	9.55 uM	Thr69 Gly72 Tyr76 Trp298 Leu300 Cys301 Asp302 Ala303 Phe329 Ala333 Ala337 Val338 Ala339 Pro340 Trp343
	11	4	−5.37	116.28 uM	Met55 Leu56 Lys57 Val58 Arg62 Val98 Ser102 Ser103 Pro126 Trp127
Ferulic acid dimer **15**	8	1-2	−5.90	47.65 uM	Leu36 Ala65 Gln68 Thr69 Gly72 Tyr76 Cys301 Asp302 Ala303 Arg306 His308 Ala337 Val338 Trp343
	25	1-2	−5.37	115.39 uM	Gln68 Thr69 Tyr76 Trp298 Leu300 Cys301 Asp302 Ala303 Phe329 Ala333 Gly336 Ala337 Val338 Ala339 Pro340
	10	4-5	−4.98	221.88 uM	Gln53 Gln54 Leu56 Lys57 Val58 Asp59 Arg62 Leu63
**NPD352**	36	1-2	−8.85	327.93 nM	Gln68 Gly72 Tyr76 Leu300 Cys301 Asp302 Ala303 His308 Phe329 Ala333 Ala337 Val338 Trp343

^a^ E.F.E.B.: Estimated Free Energy of Binding, ^b^ E.I.C., Ki: Estimated Inhibition Constant, Ki.

**Table 3:** Effect of tested compounds **1–15** in vitro, alone and in combination, on fungal growth and trichothecene (DON, 3-ADON) production by *Fusarium culmorum* FcUK99.

Treatment	^a^ Dry Fungal Biomass (Yield (mg) ± SE)	^b^ DON (Yield (ng/mL) ± SE)	
		DON	3-ADON
Control	50.36 ± 1.00	19.96 ± 2.30	80.26 ± 9.80
*p*-Coumaric acid **1**	50.14 ± 0.64	53.52 ± 9.94 *	285.11 ± 32.79 *
Caffeic acid **2**	48.56 ± 1.98	21.58 ± 1.32	66.85 ± 8.09
Ferulic acid **3**	44.20 ± 2.08	0.73 ± 0.31	1.35 ± 0.56 *
Propyl gallate **4**	20.60 ± 1.07 *	N.D.	86.20 ± 4.89
Octyl gallate **5**	0 *	N.D.	N.D.
Carvacrol **6**	52.08 ± 2.13	0.34 ± 0.22	2.11 ± 0.86 *
Thymol **7**	25.80 ± 4.50 *	N.D.	N.D.
Linalool **8**	45.52 ± 0.82	33.95 ± 1.77	81.68 ± 5.68
Geraniol **9**	44.82 ± 0.95	18.45 ± 2.86	56.26 ± 9.89
Apocynin **10**	0 *	N.D.	N.D.
Eugenol **11**	40.98 ± 1.91 *	N.D.	2.58 ± 1.68 *
Magnolol **12**	0 *	N.D.	N.D.
Honokiol **13**	0 *	N.D.	N.D.
Eugenol dimer **14**	44.12 ± 1.44	119.87 ± 12.51 *	583.15 ± 51.33 *
Ferulic acid dimer **15**	49.74 ± 1.06	14.33 ± 1.22	39.94 ± 3.14
Propyl gallate **4** + thymol **7**	28.20 ± 0.96 *	N.D.	N.D.
Magnolol **12** + thymol **7**	0 *	N.D.	N.D.
Honokiol **13** + thymol **7**	0 *	N.D.	N.D.

^a^ Dry fungal biomass (mg) and ^b^ DON, 3-ADON yields (ng/mL) are expressed as mean values (±standard error). Treatments labelled with an asterisk (*) are significantly different from the control level mean (Dunnett test). N.D.: not detected.

**Table 4:** Effect of selected compounds, alone or in combination, on disease index, grain yield and trichothecene (DON, 3-ADON) production by *Fusarium culmorum* FcUK99 in durum wheat (cv. Saragolla).

Treatment	^a^ Disease Index	^b^ Grain Yield (g)	^c^ DON (Relative Yield ± SE)	
			DON	3-ADON
Control	63.86 ± 9.50 ^a^	12.54 ± 1.46 ^a^	4528.47 ± 612.45 ^a^	79.67 ± 13.35 ^a^
Propyl gallate **4**	37.76 ± 10.49 ^a,b^	14.41 ± 1.55 ^a^	1308.49 ± 207.75 ^c,d^	16.72 ± 1.97 ^b,c^
Octyl gallate **5**	13.34 ± 4.51 ^b^	17.56 ± 0.94 ^a^	53.985 ± 37.46 ^d^	N.D.
Thymol **7**	30.0 ± 10.46 ^a,b^	15.40 ± 1.09 ^a^	3079.80 ± 546.96 ^a,b^	51.65 ± 19.63 ^a,b^
Magnolol **12**	19.98 ± 8.68 ^a,b^	15.64 ± 0.44 ^a^	1914.40 ± 181.32 ^b,c^	15.94 ± 5.46 ^b,c^
Honokiol **13**	21.10 ± 6.72 ^a,b^	17.32 ± 0.67 ^a^	207.34 ± 51.72 ^d^	4.01 ± 1.31 ^c^
Propyl gallate **4** + thymol **7**	32.20 ± 9.85 ^a,b^	15.74 ± 1.42 ^a^	19.14 ± 9.74 ^d^	4.10 ± 1.98 ^c^
Magnolol **12** + thymol **7**	36.64 ± 13.89 ^a,b^	15.74 ± 0.97 ^a^	3680.76 ± 536.69 ^a^	60.16 ± 10.93 ^a,b^
Tebuconazole	1.10 ± 1.10	17.08 ± 0.48	10.06 ± 1.33	N.D.

^a^ Disease index (0–100), ^b^ Grain yield (g) and ^c^ DON and 3-ADON (ng/g) production are expressed as mean values (±standard error). Treatments sharing the same letter are not statistically different (Tukey test). Tebuconazole has not been included in the statistical analysis to better observe the difference between the treatments. N.D.: not detected.

**Table 5:** LOD and LOQ analyte’s values.

Analyte	LOD (ng mL^−1^)	LOQ (ng mL^−1^)
DON	0.05	0.7
3-ADON	0.05	1.5
15-ADON	0.05	1.5

## Data Availability

The data presented in this study are available at https://www.mdpi.com/journal/toxins/special_issues/Fusarium_Toxins, accessed date 21 October 2021.

## Supplementary Materials

The following are available online at https://www.mdpi.com/article/10.3390/toxins13110759/s1, Table S1: H-bond interaction of tested ligands protein (TRI5-PPi) and logP of the ligands; Table S2: H-bond interaction of tested ligands-protein (TRI5-FPP) of the ligands; Figure S1: Estimated hydrophobic interactions of compounds **4**, **5**, **7** and **12** with the amino acids residues of TRI5-FPP; Table S3: Estimated hydrophobic interactions of compounds **4**, **5**, **7** and **12** with the amino acids residues of TRI5-FPP illustrated in Figure S1; Table S4: Measurement of pH in liquid Vogel’s medium amended with β-CD (3 mM) and different phenolic compounds at 0.25 mM after 0, 1, 3, 4, 5, 6, 8, 10 and 14 days (d) post inoculation with *F. culmorum* wild-type FcUK99 at 25 °C. *pKa of the carboxylic group; #pKa of the second phenolic -OH group.

## References

[1] Goswami R.S., Kistler H.C. (2004). Heading for disaster: *Fusarium graminearum* on cereal crops. Mol. Plant Pathol..

[2] Miedaner T., Cumagun C.J.R., Chakraborty S. (2008). Population genetics of three important head blight pathogens *Fusarium graminearum*, *F. pseudograminearum* and *F. culmorum*. J. Phytopathol..

[3] Scherm B., Balmas V., Spanu F., Pani G., Delogu G., Pasquali M., Migheli Q. (2013). *Fusarium culmorum*: Causal agent of foot and root rot and head blight on wheat. Mol. Plant Pathol..

[4] Haile J.K., N’Diaye A., Walkowiak S., Nilsen K.T., Clarke J.M., Kutcher H.R., Steiner B., Buerstmayr H., Pozniak C.J. (2019). *Fusarium* head blight in durum wheat: Recent status, breeding directions, and future research prospects. Phytopathology.

[5] Fernando W.D., Oghenekaro A.O., Tucker J.R., Badea A. (2021). Building on a foundation: Advances in epidemiology, resistance breeding, and forecasting research for reducing the impact of *Fusarium* head blight in wheat and barley. Can. J. Plant Pathol..

[6] Pasquali M., Migheli Q. (2014). Genetic approaches to chemotype determination in type B trichothecene producing *Fusaria*. Int. J. Food Microbiol..

[7] Foroud N.A., Baines D., Gagkaeva T.Y., Thakor N., Badea A., Steiner B., Bürstmayr M., Bürstmayr H. (2019). Trichothecenes in Cereal grains—An update. Toxins.

[8] Proctor R.H., McCormick S.P., Gutiérrez S. (2020). Genetic bases for variation in structure and biological activity of trichothecene toxins produced by diverse fungi. Appl. Microbiol. Biotechnol..

[9] Sudakin D.L. (2003). Trichothecenes in the environment: Relevance to human health. Toxicol. Lett..

[10] Zhang J., You L., Wu W., Wang X., Chrienova Z., Nepovimova E., Qinghua W., Kuca K. (2020). The neurotoxicity of trichothecenes T-2 toxin and deoxynivalenol (DON): Current status and future perspectives. Food Chem. Toxicol..

[11] Wei C.M., McLaughlin C.S. (1974). Structure-function relationship in 12, 13-epoxy trichothecenes novel inhibitors of protein-synthesis. Biochem. Biophys. Res. Commun..

[12] Desmond O.J., Manners J.M., Stephens A.E., Maclean D.J., Schenk P.M., Gardiner D.M., Munn A.L., Kazan K. (2008). The *Fusarium* mycotoxin deoxynivalenol elicits hydrogen peroxide production, programmed cell death and defence responses in wheat. Mol. Plant Pathol..

[13] Yang G.H., Jarvis B.B., Chung Y.J., Pestka J.J. (2000). Apoptosis induction by the satratoxins and other trichothecene mycotoxins: Relationship to ERK, p8 MAPK, and SAPK/JNK activation. Toxicol. Appl. Pharmacol..

[14] Spanu F., Scherm B., Camboni I., Balmas V., Pani G., Oufensou S., Macciotta N., Pasquali M., Migheli Q. (2018). *FcRav2*, a gene with a ROGDI domain involved in Fusarium head blight and crown rot on durum wheat caused by *Fusarium culmorum*. Mol. Plant Pathol..

[15] Rauwane M.E., Ogugua U.V., Kalu C.M., Ledwaba L.K., Woldesemayat A.A., Ntushelo K. (2020). Pathogenicity and virulence factors of *Fusarium graminearum* including factors discovered using next generation sequencing technologies and proteomics. Microorganisms.

[16] Hohn T.M., Beremand P. (1989). Isolation and nucleotide sequence of a sesquiterpene cyclase gene from the trichothecene-producing fungus *Fusarium sporotrichioides*. Gene.

[17] Vedula L.S., Zhao Y., Coates R.M., Koyama T., Cane D., Christianson D.W. (2007). Exploring biosynthetic diversity with trichodiene synthase. Arch. Biochem. Biophys..

[18] Vedula L.S., Jiang J., Zakharian T., Cane D.E., Christianson D.W. (2008). Structural and mechanistic analysis of trichodiene synthase using site-directed mutagenesis: Probing the catalytic function of tyrosine-295 and the asparagine-225/serine-229/glutamate-233-Mg^2+^ B motif. Arch. Biochem. Biophys..

[19] Christianson D.W. (2006). Structural biology and chemistry of the terpenoid cyclases. Chem. Rev..

[20] Rynkiewicz M.J., Cane D.E., Christianson D.W. (2002). X-ray crystal structures of D100E trichodiene synthase and its pyrophosphate complex reveal the basis for terpene product diversity. Biochemistry.

[21] Pani G., Scherm B., Azara E., Balmas V., Jahanshiri Z., Carta P., Fabbri D., Dettori M.A., Fadda A., Dessì A. (2014). Natural and natural-like phenolic inhibitors of type B trichothecene in vitro production by the wheat (*Triticum* sp.) pathogen *Fusarium culmorum*. J. Agric. Food Chem..

[22] Chala A., Weinert J., Wolf G.A. (2003). An integrated approach to the evaluation of the efficacy of fungicides against *Fusarium culmorum*, the cause of head blight of wheat. J. Phytopathol..

[23] Haidukowski M., Pascale M., Perrone G., Pancaldi D., Campagna C., Visconti A. (2005). Effect of fungicides on the development of Fusarium head blight, yield and deoxynivalenol accumulation in wheat inoculated under field conditions with *Fusarium graminearum* and *Fusarium culmorum*. J. Sci. Food Agric..

[24] Simpson D.R., Weston G.E., Turner J.A., Jennings P., Nicholson P. (2001). Differential control of head blight pathogens of wheat by fungicides and consequences for mycotoxin contamination of grain. Eur. J. Plant Pathol..

[25] Picot A., Atanasova-Pénichon V., Pons S., Marchegay G., Barreau C., Pinson-Gadais L., Roucolle J., Daveau F., Caron D., Richard-Forget F. (2013). Maize kernel antioxidants and their potential involvement in Fusarium Ear Rot resistance. J. Agric. Food Chem..

[26] Barral B., Chillet M., Minier J., Léchaudel M., Schorr-Galindo S. (2017). Evaluating the response to *Fusarium ananatum* inoculation and antifungal activity of phenolic acids in pineapple. Fungal Biol..

[27] Zhou X., Jia H., Ge X., Fengzhi W. (2018). Effects of vanillin on the community structures and abundances of *Fusarium* and *Trichoderma* spp. in cucumber seedling rhizosphere. J. Plant Interact..

[28] Pizzolitto R.P., Jacquat A.G., Usseglio V.L., Achimón F., Cuello A.E., Zygadlo J.A., Dambolena J.S. (2020). Quantitative-structure-activity relationship study to predict the antifungal activity of essential oils against *Fusarium verticillioides*. Food Control.

[29] Bakan B., Bily A.C., Melcion D., Cahagnier B., Regnault-Roger C., Philogène B.J., Richard-Molard D. (2003). Possible role of plant phenolics in the production of trichothecenes by *Fusarium graminearum* strains on different fractions of maize kernels. J. Agric. Food Chem..

[30] Boutigny A.L., Atanasova-Pénichon V., Benet M., Barreau C., Richard-Forget F. (2010). Natural phenolic acids from wheat bran inhibit *Fusarium culmorum* trichothecene biosynthesis in vitro by repressing Tri gene expression. Eur. J. Plant Pathol..

[31] Pagnussatt F.A., Del Ponte E.M., Garda-Buffon J., Badiale-Furlong E. (2014). Inhibition of *Fusarium graminearum* growth and mycotoxin production by phenolic extract from *Spirulina* sp.. Pestic. Biochem. Physiol..

[32] Pani G., Dessì A., Dallocchio R., Scherm B., Azara E., Delogu G., Migheli Q. (2016). Natural phenolic inhibitors of trichothecene biosynthesis by the wheat fungal pathogen *Fusarium culmorum*: A computational insight into the structure-activity relationship. PLoS ONE.

[33] Ferruz E., Loran S., Herrera M., Gimenez I., Bervis N., Barcena C., Carramiñana J.J., Juan T., Herrera A., Ariño A. (2016). Inhibition of *Fusarium* growth and mycotoxin production in culture medium and in maize kernels by natural phenolic acids. J. Food Prot..

[34] Schöneberg T., Martin C., Wettstein F.E., Bucheli T.D., Mascher F., Bertossa M., Musa T., Keller B., Vogelgsang S. (2016). *Fusarium* and mycotoxin spectra in Swiss barley are affected by various cropping techniques. Food Addit. Contam..

[35] Kulik T., Stuper-Szablewska K., Bilska K., Buśko M., Ostrowska-Kołodziejczak A., Załuski D., Perkowski J. (2017). Trans-cinnamic and chlorogenic acids affect the secondary metabolic profiles and ergosterol biosynthesis by *Fusarium culmorum* and *F. graminearum* sensu stricto. Toxins.

[36] Kulik T., Stuper-Szablewska K., Bilska K., Buśko M., Ostrowska-Kołodziejczak A., Załuski D., Perkowski J. (2017). Sinapic acid affects phenolic and trichothecene profiles of *F. culmorum* and *F. graminearum* sensu stricto. Toxins.

[37] Scaglioni P.T., Scarpino V., Marinaccio F., Vanara F., Badiale-Furlong E., Blandino M. (2019). Impact of microalgal phenolic extracts on the control of *Fusarium graminearum* and deoxynivalenol contamination in wheat. World Mycotoxin J..

[38] Oufensou S., Scherm B., Pani G., Balmas V., Fabbri D., Dettori M.A., Carta P., Malbrán I., Migheli Q., Delogu G. (2019). Honokiol, magnolol and its monoacetyl derivative show strong antifungal effect on Fusarium isolates of clinical relevance. PLoS ONE.

[39] Oufensou S., Balmas V., Azara E., Fabbri D., Dettori M.A., Schüller C., Zehetbauer F., Strauss J., Delogu G., Migheli Q. (2020). Naturally occurring phenols modulate vegetative growth and deoxynivalenol biosynthesis in *Fusarium graminearum*. ACS Omega.

[40] Malbrán I., Mourelos C.A., Pardi M., Oufensou S., Balmas V., Delogu G., Migheli Q., Lori G.A., Juárez M.P., Girotti J.A. (2020). Commercially available natural inhibitors of trichothecene production in Fusarium graminearum: A strategy to manage Fusarium head blight of wheat. Crop. Prot..

[41] Montibus M., Vitrac X., Coma V., Loron A., Pinson-Gadais L., Ferrer N., Verdal-Bonnin M.N., Gabaston J., Waffo-Téguo P., Richard-Forget F. (2021). Screening of wood/forest and vine by-products as sources of new drugs for sustainable strategies to control *Fusarium graminearum* and the production of mycotoxins. Molecules.

[42] Boutigny A.L., Barreau C., Atanasova-Pénichon V., Verdal-Bonnin M.N., Pinson-Gadais L., Richard-Forget F. (2009). Ferulic acid, an efficient inhibitor of type B trichothecene biosynthesis and *Tri* gene expression in *Fusarium* liquid cultures. Mycol. Res..

[43] Dambolena J.S., López A.G., Meriles J.M., Rubinstein H.R., Zygadlo J.A. (2012). Inhibitory effect of 10 natural phenolic compounds on *Fusarium verticillioides*. A structure–property–activity relationship study. Food Control.

[44] Chen C., Long L., Zhang F., Chen Q., Chen C., Yu X., Liu Q., Bao J., Long Z. (2018). Antifungal activity, main active components and mechanism of *Curcuma longa* extract against *Fusarium graminearum*. PLoS ONE.

[45] Zhang M., Ge J., Yu X. (2018). Transcriptome analysis reveals the mechanism of fungicidal of thymol against *Fusarium oxysporum* f. sp. niveum. Curr. Microbiol..

[46] Boddu J., Cho S., Muehlbauer G.J. (2007). Transcriptome analysis of trichothecene-induced gene expression in Barley. Mol. Plant-Microbe Interact..

[47] Boutigny A.L., Richard-Forget F., Barreau C. (2008). Natural mechanisms for cereal resistance to the accumulation of *Fusarium* trichothecenes. Eur. J. Plant Pathol..

[48] Atanasova-Pénichon V., Barreauand C., Richard-Forget F. (2016). Antioxidant secondary metabolites in cereals: Potential involvement in resistance to *Fusarium* and mycotoxin accumulation. Front. Microbiol..

[49] Hadjout S., Chéreau S., Atanasova-Pénichon V., Marchegay G., Mekliche L., Boureghda H., Barreau C., Touati-Hattab S., Bouznad Z., Richard-Forget F. (2017). Phenotypic and biochemical characterization of new advanced durum wheat breeding lines from Algeria that show resistance to Fusarium head blight and to mycotoxin accumulation. J. Plant Pathol..

[50] Kimura M., Tokai T., Takahashi-Ando N., Ohsato S., Fujimura M. (2007). Molecular and genetic studies of *Fusarium* trichothecene biosynthesis: Pathways, genes and evolution. Biosci. Biotechnol. Biochem..

[51] Rynkiewicz M.J., Cane D.E., Christianson D.W. (2001). Structure of trichodiene synthase from *Fusarium sporotrichioides* provides mechanistic inferences on the terpene cyclization cascade. Proc. Natl. Acad. Sci. USA.

[52] Maeda K., Nakajima Y., Motoyama T., Kondoh Y., Kawamura T., Kanamaru K., Ohsato S., Nishiuchi T., Yoshida M., Osada H. (2017). Identification of a trichothecene production inhibitor by chemical array and library screening using trichodiene synthase as a target protein. Pestic. Biochem. Physiol..

[53] Wolf V.G., Bonacorsi C., Raddi M.S.G., da Fonseca L.M., Ximenes V.F. (2017). Octyl gallate, a food additive with potential beneficial properties to treat Helicobacter pylori infection. Food Funct..

[54] Singh S., Fatima Z., Hameed S. (2020). Octyl gallate reduces ABC multidrug transporter CaCdr1p expression and leads to its mislocalisation in azole-resistant clinical isolates of *Candida albicans*. J. Glob. Antimicrob. Resist..

[55] Kim J.H., Chan K.L., Cheng L.W. (2018). Octyl gallate as an intervention catalyst to augment antifungal efficacy of Caspofungin. Multidiscip. Sci. J..

[56] Chen Y.H., Lu M.H., Guo D.S., Zhai Y.Y., Miao D., Yue J.Y., Yuan C.H., Zhao M.M., An D.R. (2019). Antifungal effect of magnolol and honokiol from *Magnolia officinalis* on *Alternaria alternata* causing tobacco brown spot. Molecules.

[57] Zhao C., Liu Z.Q. (2011). Comparison of antioxidant abilities of magnolol and honokiol to scavenge radicals and to protect DNA. Biochimie.

[58] Amorati R., Zotova J., Baschieri A., Valgimigli L. (2015). Antioxidant activity of magnolol and honokiol: Kinetic and mechanistic investigations of their reaction with peroxyl radicals. J. Org. Chem..

[59] Rachitha P., Krupashree K., Jayashree G.V., Gopalan N., Khanum F. (2017). Growth Inhibition and morphological alteration of *Fusarium sporotrichioides* by *Mentha piperita* essential oil. Pharmacogn. Res..

[60] Liu D., Pan Y., Li K., Li D., Li P., Gao Z. (2020). Proteomics reveals the mechanism underlying the inhibition of Phytophthora sojae by propyl gallate. J. Agric. Food Chem..

[61] Takai E., Hirano A., Shiraki K. (2011). Effects of alkyl chain length of gallate on self-association and membrane binding. J. Biochem..

[62] Hyldgaard M., Mygind T., Meyer R.L. (2012). Essential oils in food preservation: Mode of action, synergies, and interactions with food matrix components. Front. Microbiol..

[63] Gao T., Zhou H., Zhou W., Hu L., Chen J., Shi Z. (2016). The fungicidal activity of thymol against *Fusarium graminearum* via inducing lipid peroxidation and disrupting ergosterol biosynthesis. Molecules.

[64] Divband K., Shokri H., Khosravi A.R. (2017). Down-regulatory effect of *Thymus vulgaris* L. on growth and *Tri4* gene expression in *Fusarium oxysporum* strains. Microb. Pathog..

[65] Gutiérrez-Fernández J., García-Armesto M.R., Álvarez-Alonso R., del Valle P., de Arriaga D., Rúa J. (2013). Antimicrobial activity of binary combinations of natural and synthetic phenolic antioxidants against *Enterococcus faecalis*. J. Dairy Sci..

[66] de Castro R.D., de Souza T.M., Bezerra L.M., Ferreira G.L., Costa E.M., Cavalcanti A.L. (2015). Antifungal activity and mode of action of thymol and its synergism with nystatin against *Candida* species involved with infections in the oral cavity: An in vitro study. BMC Complement. Altern. Med..

[67] Sun L., Liao K., Hang C., Wang D. (2017). Honokiol induces reactive oxygen species-mediated apoptosis in *Candida albicans* through mitochondrial dysfunction. PLoS ONE.

[68] Sun L., Liao K., Wang D. (2015). Effect of magnolol and honokiol on adhesion, yeast-hyphal transition, and formation of biofilm by *Candida albicans*. PLoS ONE.

[69] Wang Z. (2017). Plant-derived antifungal compounds trigger a common transcriptional response. Inf. Genet. Evol..

[70] Wang Z., Shen Y. (2016). Antifungal compound honokiol triggers oxidative stress responsive signalling pathway and modulates central carbon metabolism. Mycology.

[71] Lowe R.G.T., Allwood J.W., Galster A., Urban M., Daudi A., Canning C., Ward J.L., Beale M.H., Hammond-Kosack K.E. (2010). A combined ^H^NMR and ESI-MS analysis to understand the basal metabolism of plant pathogenic *Fusarium* species. Mol. Plant-Microbe Interact..

[72] De Farias Dias A. (1988). An improved high yield synthesis of dehydrodieugenol. Phytochemistry.

[73] Russell W.R., Scobbie L., Chesson A. (2005). Structural modification of phenylpropanoid-derived compounds and the effects on their participation in redox processes. Bioorganic Med. Chem..

[74] Vogel H.J. (1956). A convenient growth medium for Neurospora (Med N). Microbiol. Genet. Bull..

[75] Laskowski R.A., Swindells M.B. (2011). LigPlot+: Multiple ligand-protein interaction diagrams for drug discovery. J. Chem. Inf. Model..

