# Supplementary material for: Molecular Docking and Comparative Inhibitory Efficacy of Naturally Occurring Compounds on Vegetative Growth and Deoxynivalenol Biosynthesis in Fusarium culmorum
Source: Toxins (Basel). 2021 Oct 26;13(11):759. doi: 10.3390/toxins13110759 (PMC8623340; doi:10.3390/toxins13110759)
Supplement: Supplementary file 1 [file toxins-13-00759-s001.zip › toxins-1380679-supplementary.pdf]

toxins

MDPI

Supplementary Information

Molecular Docking and Comparative Inhibitory Efficacy of Naturally Occurring Compounds on Vegetative Growth and Deoxynivalenol Biosynthesis in *Fusarium Culmorum*

Safa Oufensou [1,3,*], Alessandro Dessì [2], Roberto Dallocchio [2], Virgilio Balmas [1], Emanuela Azara [2], Paola Carta [2], Quirico Migheli [1,3] and Giovanna Delogu [2]

[1] Dipartimento di Agraria, Università degli Studi di Sassari, Via E. De Nicola 9, I-07100 Sassari, Italy; balmas@uniss.it (V.B.); qmigheli@uniss.it (Q.M.)
[2] Istituto CNR di Chimica Biomolecolare, Traversa La Crucca 3, I-07100 Sassari, Italy; alessandro.dessi@cnr.it, (A.D.); robertonico.dallocchio@cnr.it, (R.D.); emanuelagigliola.azara@cnr.it, (E.A.); paola.carta@cnr.it, (P.C.); giovanna.delogu@icb.cnr.it (G.D.)
[3] Nucleo di Ricerca sulla Desertificazione, Università degli Studi di Sassari, Via E. De Nicola 9, 07100 Sassari, Italy
* Correspondence: soufensou@uniss.it

Table S1. H-bond interaction of tested ligands-protein (TRI5-PPi) and logP of the ligands.

					**PPi**			
					**Hydrogen (H) Bond Interactions**			
**Pose**	**Tested Ligands**	**%**	**Sites**	**H-bond**	**Ligand Atom**	**Protein Atom**	**Distance (Å) [d]**	**LogP**
1	*p*-Coumaric acid **1**	75	c.d. [a]	2	H13(HD) [b]	Pro178:O(OA)	2.474	1.54
					O12(OA) [c]	Asn246:2HD2(HD)	2.039	
4		4	3	6	H13(HD)	Met1:O(OA)	2.081	
					O12(OA)	Met1:HN3(HD)	1.616	
					H13(HD)	Asp235:OD1(OA)	2.422	
					O10(OA)	Lys313:HZ2(HD)	**1.902**	
					O11(OA)	Lys313:HZ2(HD)	**2.585**	
					O11(OA)	Lys313:HZ3(HD)	**1.848**	
5		11	3	5	O10(OA)	Thr6:HG1(HD)	2.515	
					H13(HD)	Tyr231:O(OA)	1.895	
					O12(OA)	Asp235:HN(HD)	2.221	
					O10(OA)	Lys313:HZ2(HD)	**1.852**	
					O11(OA)	Lys313:HZ3(HD)	**1.962**	
1	Caffeic acid **2**	39	c.d.	3	H13(HD)	Ile241:O(OA)	2.290	1.15
					O12(OA)	Asn246:1HD2(HD)	**1.945**	
					O14(OA)	Asn246:2HD2(HD)	**2.179**	
5		16	4	6	H13(HD)	Met55:O(OA)	**1.997**	
					H15(HD)	Met55:O(OA)	**2.216**	
					O12(OA)	Lys57:HN(HD)	2.047	
					O11(OA)	Arg62:1HH1(HD)	**2.422**	
					O11(OA)	Arg62:1HH2(HD)	**2.336**	
					O10(OA)	Ser103:HN(HD)	2.117	
9		11	3	6	H13(HD)	Met1:O(OA)	**2.019**	
					O14(OA)	Met1:HN3(HD)	**1.856**	
					H15(HD)	Met1:O(OA)	**2.105**	
					H13(HD)	Glu2:O(OA)	2.523	
					O10(OA)	Lys313:HZ2(HD)	**2.038**	

					O11(OA)	Lys313:HZ3(HD)	**1.948**	
1	Ferulic acid **3** [32]	2	c.d.	5	O12(OA)	Asn185:2HD2(HD	2.118	1.42
					H15(HD)	Asp226:OD1(OA)	1.887	
					O11(OA)	Arg238:HE(HD)	**2.299**	
					O11(OA)	Arg238:1HH2(HD)	**2.196**	
					O14(OA)	Leu243:HN(HD)	1.985	
2		6	c.d.	0	--------------	----------------	-----------	
5		13	3	4	H15(HD)	Glu2:O(OA)	2.023	
					O14(OA)	Phe4:HN(HD)	2.093	
					O10(OA)	Asp235:HN(HD)	1.925	
					O11(OA)	Arg306:1HH2(HD)	1.779	
6		8	4	5	H15(HD)	Met55:O(OA)	2.269	
					O12(OA)	Lys57:HN(HD)	2.147	
					O10(OA)	Arg62:1HH1(HD)	**1.929**	
					O10(OA)	Arg62:1HH2(HD)	**2.235**	
					O11(OA)	Ser103:HN(HD)	2.208	
7		14	3	7	O11(OA)	Thr6:HG1(HD)	2.592	
					H15(HD)	Tyr231:O(OA)	1.860	
					O14(OA)	Asp235:HN(HD)	2.276	
					O12(OA)	Arg306:1HH1(HD)	**2.481**	
					O12(OA)	Arg306:1HH2(HD)	**2.391**	
					O10(OA)	Lys313:HZ3(HD)	**1.946**	
					O11(OA)	Lys313:HZ2(HD)	**1.809**	
9		19	5	3	O14(OA)	Gln53:2HE2(HD)	2.271	
					O10(OA)	Lys57:HZ3(HD)	**2.483**	
					O11(OA)	Lys57:HZ2(HD)	**1.718**	
1	Propyl gallate **4** [32]	40	1	7	O8(OA)	Gln68:1HE2(HD)	**2.243**	1.51
					O9(OA)	Gln68:1HE2(HD)	**2.280**	
					H18(HD)	Tyr76:OH(OA)	2.395	
					H16(HD)	Asp302:OD2(OA)	2.086	
					H14(HD)	Gly336:O(OA)	**2.134**	
					H18(HD)	Gly336:O(OA)	**2.244**	
					O13(OA)	Val338:HN(HD)	1.932	
3		13	2	5	H14(HD)	Asp302:O(OA)	**1.926**	
					H18(HD)	Asp302:O(OA)	**2.005**	
					H18(HD)	Ala303:O(OA)	2.591	
					O17(OA)	Arg306:HN(HD)	2.253	
					O13(OA)	Leu307:HN(HD)	1.979	
1	Octyl gallate **5**	12	2-1	4	H21(HD)	Asp302:O(OA)	**2.109**	3.60
					H23(HD)	Asp302:O(OA)	**2.136**	
					O22(OA)	Arg306:HN(HD)	2.404	
					O20(OA)	Leu307:HN(HD)	2.071	
2		9	1	6	O8(OA)	Gln68:1HE2(HD)	2.207	
					O22(OA)	Tyr76:HH(HD)	**2.436**	
					H23(HD)	Tyr76:OH(OA)	**2.237**	
					H19(HD)	Gly336:O(OA)	**1.938**	
					H23(HD)	Gly336:O(OA)	**2.060**	
					O18(OA)	Val338:HN(HD)	1.839	
3		16	1-2	5	H19(HD)	Gln68:O(OA)	2.039	

					H21(HD)	Asp302:OD2(OA)	**2.210**	
					H23(HD)	Asp302:OD2(OA)	**2.081**	
					O20(OA)	Ala303:HN(HD)	1.918	
					O8(OA)	Val338:HN(HD)	1.784	
5		5	1-2	7	O20(OA)	Tyr76:HH(HD)	**2.553**	
					H21(HD)	Tyr76:OH(OA)	**2.203**	
					H23(HD)	Tyr76:OH(OA)	**1.898**	
					O8(OA)	Ala303:HN(HD)	**2.045**	
					O9(OA)	Ala303:HN(HD)	**2.325**	
					H21(HD)	Gly336:O(OA)	2.153	
					O20(OA)	Val338:HN(HD)	1.837	
2	Carvacrol **6**	48	1	2	O11(OA)	Val338:HN(HD)	1.888	3.37
					H12(HD)	Gly336:O(OA)	1.870	
3		21	2	2	O11(OA)	Leu107:HN(HD)	1.992	
					H12(HD)	Asp302:O(OA)	2.168	
4		11	c.d.	2	O11(OA)	Thr96:HG1(HD)	1.937	
					H12(HD)	Tyr93:O(OA)	1.910	
6		16	4	2	O11(OA)	Lys57:HN(HD)	1.948	
					H12(HD)	Met55:O(OA)	2.087	
1	Thymol **7**	5	c.d.	1	H12(HD)	Asp266:OD1(OA)	1.961	3.37
2		20	4	2	O11(OA)	Lys57:HN(HD)	1.825	
					H12(HD)	Met55:O(OA)	1.969	
3		58	1	2	O11(OA)	Val338:HN(HD)	**1.844**	
					H12(HD)	Val338:O(OA)	**1.855**	
4		10	c.d.	2	O11(OA)	Thr96:HG1(HD)	2.248	
					H12(HD)	Tyr93:O(OA)	1.807	
1	*R*-Linalool **8**	36	1	2	H12(HD)	Gly336:O(OA)	1.877	2.55
					O11(OA)	Val338:HN(HD)	1.902	
2		21	c.d.	2	H12(HD)	Tyr93:O(OA)	1.867	
					O11(OA)	Thr96:HG1(HD)	1.947	
3		12	1	2	O11(OA)	Trp298:HE1(HD)	2.235	
					H12(HD)	Asp302:O(OA)	1.741	
5		10	4	2	H12(HD)	Met55:O(OA)	1.806	
					O11(OA)	Lys57:HN(HD)	1.786	
2	*S*-Linalool **8**	11	4	2	H6(HD)	Met55:O(OA)	1.761	2.55
					O5(OA)	Lys57:HN(HD)	1.785	
3		36	1	2	H6(HD)	Gly336:O(OA)	1.830	
					O5(OA)	Val338:HN(HD)	2.140	
4		27	c.d.	2	H6(HD)	Tyr93:O(OA)	1.904	
					O5(OA)	Thr96:HG1(HD)	1.951	
5		17	1	2	O5(OA)	Trp298:HE1(HD)	2.378	
					H6(HD)	Asp302:OD2(OA)	1.760	
1	Geraniol **9**	2	c.d.	1	H12(HD)	Asp239:OD1(OA)	1.796	2.49
2		12	2	2	H12(HD)	Asp302:O(OA)	1.822	
					O11(OA)	Leu307:HN(HD)	1.966	
3		47	1	2	H12(HD)	Gly336:O(OA)	1.795	
					O11(OA)	Val338:HN(HD)	1.844	
4		16	c.d.	2	O11(OA)	Arg182:2HH2(HD)	1.980	
					H12(HD)	PPi700:O3(OA)	1.825	

1	Apocynin **10** [32]	4	c.d.	3	O10(OA)	Asn185:2HD2(HD)	2.087	0.83
					H13(HD)	Asp226:OD1(OA)	1.828	
					O12(OA)	Leu243:HN(HD)	1.846	
2		60	1	4	O9(OA)	Gln68:1HE2(HD)	2.163	
					O12(OA)	Tyr76:HH(HD)	2.386	
					H13(HD)	Gly336:O(OA)	1.977	
					O10(OA)	Val338:HN(HD)	2.043	
4		11	1	3	H13(HD)	Gln68:O(OA)	2.172	
					O12(OA)	Trp298:HE1(HD)	2.305	
					O9(OA)	Val338:HN(HD)	1.822	
1	Eugenol **11** [32]	3	c.d.	3	O10(OA)	Asn185:2HD2(HD)	1.919	2.57
					H13(HD)	Asp226:OD1(OA)	1.819	
					O12(OA)	Leu243:HN(HD)	2.318	
2		31	2	1	H13(HD)	Leu300:O(OA)	2.003	
3		19	2	2	H13(HD)	Asp302:O(OA)	2.204	
					O10(OA)	Leu307:HN(HD)	1.977	
4		22	1	2	H13(HD)	Gly336:O(OA)	2.031	
					O12(OA)	Val338:HN(HD)	1.845	
1	Magnolol **12** [32]	29	1-2	3	O18(OA)	Tyr76:HH(HD)	2.482	5.03
					H19(HD)	Gly336:O(OA)	1.907	
					H17(HD)	Val338:O(OA)	1.889	
3		11	1-2	3	H17(HD)	Cys301:O(OA)	2.167	
					O16(OA)	Ala303:HN(HD)	2.412	
					H19(HD)	Val338:O(OA)	1.993	
10		14	4	4	O16(OA)	Arg62:1HH2(HD)	1.906	
					H17(HD)	Ser102:OG(OA)	**2.270**	
					H19(HD)	Ser102:OG(OA)	**1.880**	
					O18(OA)	Ser103:HN(HD)	2.091	
1	Honokiol **13**	25	1-2	3	O21(OA)	Trp298:HE1(HD)	2.358	5.03
					H22(HD)	Asp302:OD2(OA)	2.006	
					H14(HD)	Val338:O(OA)	1.914	
2		6	2	4	H14(HD)	Cys301:O(OA)	1.884	
					H22(HD)	Asp302:O(OA)	1.825	
					O21(OA)	Arg306:HN(HD)	2.466	
					O21(OA)	Leu307:HN(HD)	2.142	
3		24	1-2	3	H14(HD)	Asp302:OD2(OA)	1.735	
					H22(HD)	Gly336:O(OA)	2.071	
					O21(OA)	Val338:HN(HD)	1.638	
4		16	1-2	3	H22(HD)	Asp302:OD2(OA)	2.066	
					O13(OA)	Val338:HN(HD)	**1.835**	
					H14(HD)	Val338:O(OA)	**1.962**	
1	Eugenol dimer **14** [32]	14	1-2	3	O15(OA)	Gln68:1HE2(HD)	2.099	4.78
					O20(OA)	Val338:HN(HD)	2.176	
					H21(HD)	Val338:O(OA)	2.163	
2		28	1-2	2	O25(OA)	Ala303:HN(HD)	2.166	
					H14(HD)	Val338:O(OA)	2.187	
4		10	1-2	3	H21(HD)	Tyr76:OH(OA)	2.458	
					H21(HD)	Gln336:O(OA)	2.141	
					O20(OA)	Val338:HN(HD)	1.991	

7		14	4	4	H14(HD)	Lys57:O(OA)	2.019	
					O13(OA)	Arg62:1HH2(HD)	1.881	
					O20(OA)	Ser103:HN(HD)	**1.827**	
					H21(HD	Ser103:OG(OA)	**2.155**	
1	Ferulic acid dimer **15**	4	1-2	4	O20(OA)	Gln68:1HE2(HD)	**2.180**	2.47
					O20(OA)	Gln68:H23(HD)	**1.971**	
					O16(OA)	Thr69:HG1(HD)	2.499	
					O16(OA)	Arg304:2HH1(HD)	1.986	
5		26	2-1	4	O27(OA)	Gln68:1HE2(HD)	2.596	
					H21(HD)	Cys301:O(OA)	2.160	
					H23(HD)	Gln336:O(OA)	2.214	
					O22(OA)	Val338:HN(HD)	2.173	
1	**NPD352**	6	1-2	2	H27(HD)	Cys301:O(OA)	2.170	4.21
					O31(OA)	Ala303:HN(HD)	1.976	
6		22	1-2	1	H41(HD	Asp302:OD2(OA)	2.148	

[a] c.d.: catalytic domain, [b] Hydrogen donor, [c] Oxygen acceptor, [d] Cross-bridge H-bond interactions with the same aa are listed in bold.

Table S2. H-bond interaction of tested ligands-protein (TRI5-FPP) of the ligands.

FPP<br>Hydrogen (H) Bond Interactions							
**Pose**	**Tested Ligands**	**%**	**Sites**	**H-bond**	**Ligand Atom**	**Protein Atom**	**Distance (Å)** [c]
1	*p*-Coumaric acid **1**	27	3	5	O12(OA) [a]	Met1:HN3(HD)	**1.616**
					H13(HD) [b]	Met1:O(OA)	**2.078**
					H13(HD)	Asp235:OD1(OA)	2.426
					O10(OA)	Lys313:HZ2(HD)	**1.910**
					O11(OA)	Lys313:HZ3(HD)	**1.897**
2		45	3	5	O10(OA)	Thr6:HG1(HD)	2.569
					H13(HD)	Tyr231:O(OA)	1.905
					O12(OA)	Asp235:HN(HD)	2.252
					O10(OA)	Lys313:HZ2(HD)	**1.817**
					O11(OA)	Lys313:HZ3(HD)	**1.948**
4		22	5	4	O12(OA)	Gln53:2HE2(HD)	2.245
					O10(OA)	Lys57:HZ2(HD)	**1.705**
					O11(OA)	Lys57:HZ2(HD)	**2.564**
					O11(OA)	Lys57:HZ2(HD)	**2.388**
1	Caffeic acid **2**	20	4	6	H15(HD)	Met55:O(OA)	**2.016**
					H13(HD)	Met55:O(OA)	**1.881**
					O12(OA)	Lys57:HN(HD)	2.165
					O11(OA)	Arg62:1HH1(HD)	**2.328**
					O11(OA)	Arg62:1HH2(HD)	**1.916**
					O10(OA)	Ser103:HN(HD)	2.146
3		15	5	5	O14(OA)	Gln53:2HE2(HD)	**2.220**
					H13(HD)	Gln53:OE1(OA)	**2.203**
					O10(OA)	Lys57:HZ2(HD)	**2.584**
					O10(OA)	Lys57:HZ3(HD)	**2.399**
					O11(OA)	Lys57:HZ2(HD)	**1.706**
4		25	3	6	H13(HD)	Met1:O(OA)	**1.997**
					O14(OA)	Met1:HN3(HD)	**1.865**
					H15(HD)	Met1:O(OA)	**2.083**
					H13(HD)	Glu2:O(OA)	2.539
					O10(OA)	Lys313:HZ2(HD)	**2.055**
					O11(OA)	Lys313:HZ3(HD)	**1.954**
1	Ferulic acid **3**	16	3	4	O14(OA)	Met1:HN3(HD)	**1.815**
					H15(HD)	Met1:O(OA)	**2.138**
					O11(OA)	Lys313:HZ2(HD)	**1.752**
					O10(OA)	Lys313:HZ3(HD)	**1.899**
2		9	4	5	H15(HD)	Met55:O(OA)	2.287
					O12(OA)	Lys57:HN(HD)	2.126
					O10(OA)	Arg62:1HH1(HD)	**1.957**
					O10(OA)	Arg62:1HH2(HD)	**2.288**
					O11(OA)	Ser103:HN(HD)	2.148
3		23	5	4	O14(OA)	Gln53:2HE2(HD)	2.247
					O10(OA)	Lys57:HZ2(HD)	**2.561**
					O10(OA)	Lys57:HZ3(HD)	**2.395**
					O11(OA)	Lys57:HZ2(HD)	**1.703**

4		8	3	4	H15(HD)	Glu2:O(OA)	2.161
					O12(OA)	Phe4:HN(HD)	1.874
					O11(OA)	Asp235:HN(HD)	1.911
					O10(OA)	Arg306:2HH2(HD)	1.696
7		19	3	6	O11(OA)	Thr6:HG1(HD)	2.471
					O14(OA)	Asp235:HN(HD)	2.243
					O12(OA)	Arg306:1HH2(HD)	**2.361**
					O12(OA)	Arg306:2HH2(HD)	**2.479**
					O10(OA)	Lys313:HZ3(HD)	**1.987**
					O11(OA)	Lys313:HZ2(HD)	**1.862**
10		9	2	2	H15(HD)	Asp302:O(OA)	1.997
					O14(OA)	Leu307:HN(HD)	1.777
1	Propyl gallate **4**	2	1	5	O13(OA)	Gln68:1HE2(HD)	2.187
					H16(HD)	Asp302:OD2(OA)	**1.758**
					H18(HD)	Asp302:OD2(OA)	**1.983**
					O9(OA)	Val338:HN(HD)	2.158
					O8(OA)	Trp343:HE1(HD)	2.050
2		39	1	6	O8(OA)	Gln68:1HE2(HD)	2.305
					O17(OA)	Tyr76:HH(HD)	**2.364**
					H18(HD)	Tyr76:OH(OA)	**2.563**
					H14(HD)	Gly336:O(OA)	**2.171**
					H18(HD)	Gly336:O(OA)	**1.838**
					O13(OA)	Val338:HN(HD)	1.941
3		16	2	5	H14(HD)	Asp302:O(OA)	**2.042**
					H18(HD)	Asp302:O(OA)	**2.012**
					H18(HD)	Ala303:O(OA)	2.566
					O17(OA)	Arg306:HN(HD)	2.257
					O13(OA)	Leu307:HN(HD)	1.934
6		7	1-2	4	O17(OA)	Trp298:HE1(HD)	2.583
					H16(HD)	Asp302:OD2(OA)	**1.838**
					H18(HD)	Asp302:OD2(OA)	**1.947**
					O8(OA)	Val338:HN(HD)	1.731
11		12	4	6	O8(OA)	Asp59:HN(HD)	1.816
					O15(OA)	Arg62:1HH2(HD)	2.166
					H16(HD)	Ser102:OG(OA)	**1.824**
					H18(HD)	Ser102:OG(OA)	**1.820**
					H14(HD)	Ser103:OG(OA)	**2.102**
					O17(OA)	Ser103:HN(HD)	**1.886**
1	Octyl gallate **5**	12	2-1	4	H21(HD)	Asp302:O(OA)	**2.091**
					H23(HD)	Asp302:O(OA)	**2.005**
					O22(OA)	Arg306:HN(HD)	2.258
					O20(OA)	Leu307:HN(HD)	2.114
2		15	1-2	4	O22(OA)	Trp298:HE1(HD)	2.466
					H23(HD)	Asp302:OD2(OA)	**1.852**
					H19(HD)	Asp302:OD2(OA)	**1.793**
					O8(OA)	Val338:HN(HD)	1.734
3		10	1	6	O8(OA)	Gln68:1HE2(HD)	2.302
					O22(OA)	Tyr76:HH(HD)	**2.201**
					H23(HD)	Tyr76:OH(OA)	**2.543**

					H23(HD)	Gly336:O(OA)	**1.800**
					H21(HD)	Gly336:O(OA)	**2.138**
					O20(OA)	Val338:HN(HD)	1.923
6		9	2-1	4	H19(HD)	Leu300:O(OA)	**1.655**
					H23(HD)	Leu300:O(OA)	**1.726**
					H21(HD)	Asp302:O(OA)	2.125
					O20(OA)	Leu307:HN(HD)	1.796
2	Carvacrol **6**	62	1	2	H12(HD)	Gly336:O(OA)	1.875
					O11(OA)	Val338:HN(HD)	1.885
3		24	2	2	H12(HD)	Asp302:O(OA)	2.171
					O11(OA)	Leu307:HN(HD)	1.981
5		9	4	2	H12(HD)	Met55:O(OA)	2.086
					O11(OA)	Lys57:HN(HD)	1.870
1	Thymol **7**	18	4	2	H12(HD)	Met55:O(OA)	1.973
					O11(OA)	Lys57:HN(HD)	1.792
2		72	1	2	O11(OA)	Val338:HN(HD)	**1.833**
					H12(HD)	Val338:O(OA)	**1.844**
3		6	1	2	H12(HD)	Gly336:O(OA)	1.902
					O11(OA)	Val338:HN(HD)	1.750
1	*R*-Linalool **8**	11	1	2	O11(OA)	Trp298:HE1(HD)	2.257
					H12(HD)	Asp302:O(OA)	1.755
2		60	1	2	H12(HD)	Gly336:O(OA)	1.810
					O11(OA)	Val338:HN(HD)	1.865
3		9	4	2	O11(OA)	Met55:O(OA)	1.811
					H12(HD)	Lys57:HN(HD)	1.799
4		9	1	2	O11(OA)	Trp298:HE1(HD)	2.215
					H12(HD)	Asp302:OD2(OA)	1.773
1	*S*-Linalool **8**	8	4	2	H6(HD)	Met55:O(OA)	1.841
					O5(OA)	Lys57:HN(HD)	1.742
2		55	1	2	H6(HD)	Gly336:O(OA)	1.804
					O5(OA)	Val338:HN(HD)	2.046
3		25	1	2	O5(OA)	Trp298:HE1(HD)	2.369
					H6(HD)	Asp302:OD2(OA)	1.758
1	Geraniol **9**	13	2	2	H12(HD)	Asp302:O(OA)	1.844
					O11(OA)	Leu307:HN(HD)	1.949
2		55	1	2	H12(HD)	Gly336:O(OA)	1.890
					O11(OA)	Val338:HN(HD)	1.782
3		7	1	2	O11(OA)	Asp302:O(OA)	1.948
					H12(HD)	Ala303:HN(HD)	2.129
4		12	4	2	H12(HD)	Ser102:OG(OA)	1.831
					O11(OA)	Ser103:HN(HD)	2.132
1	Apocynin **10**	67	1	4	O9(OA)	Gln68:1HE2(HD)	2.175
					O12(OA)	Tyr76:HH(HD)	2.381
					H13(HD)	Gly336:O(OA)	1.909
					O10(OA)	Val338:HN(HD)	2.028
2		12	1	3	H13(HD)	Gln68:O(OA)	2.178
					O12(OA)	Trp298:HE1(HD)	2.307
					O9(OA)	Val338:HN(HD)	1.821
3		11	4	3	O9(OA)	Lys57:HN(HD)	1.841

					O12(OA)	Arg62:1HH2(HD)	1.935
					H13(HD)	Ser102:OG(OA)	2.140
1	Eugenol **11**	27	2	2	H13(HD)	Asp302:O(OA)	2.189
					O10(OA)	Leu307:HN(HD)	2.021
2		23	2	1	H13(HD)	Leu300:O(OA)	1.978
3		32	1	2	H13(HD)	Gly336:O(OA)	1.938
					O12(OA)	Val338:HN(HD)	1.854
6		12	4	2	O12(OA)	Arg62:1HH2(HD)	1.979
					H13(HD)	Ser102:OG(OA)	2.210
1	Magnolol **12**	38	1-2	4	O16(OA)	Tyr76:HH(HD)	2.334
					H17(HD)	Gly336:O(OA)	1.959
					O16(OA)	Val338:HN(HD)	**2.165**
					H19(HD)	Val338:O(OA)	**1.892**
3		14	1-2	4	H19(HD)	Tyr76:HH(HD)	2.569
					H17(HD)	Gly336:O(OA)	**1.992**
					H19(HD)	Gly336:O(OA)	**2.206**
					O16(OA)	Val338:O(OA)	1.627
4		10	1-2	2	H17(HD)	Val338:O(OA)	**1.932**
					H19(HD)	Val338:O(OA)	**1.795**
7		17	4	4	O16(OA)	Arg62:1HH2(HD)	1.865
					H17(HD)	Ser102:OG(OA)	**2.161**
					H19(HD)	Ser102:OG(OA)	**1.846**
					O18(OA)	Ser103:HN(HD)	1.849
1	Honokiol **13**	23	1-2	3	O21(OA)	Trp298:HE1(HD)	2.354
					H22(HD)	Asp302:OD2(OA)	2.003
					H14(HD)	Val338:O(OA)	1.853
2		13	2	4	H14(HD)	Cys301:O(OA)	1.945
					H22(HD)	Asp302:O(OA)	1.804
					O21(OA)	Arg306:HN(HD)	2.485
					O21(OA)	Leu307:HN(HD)	2.096
3		23	1	3	H14(HD)	Asp302:OD2(OA)	1.734
					H22(HD)	Gly336:O(OA)	1.979
					O21(OA)	Val338:HN(HD)	1.628
5		15	1-2	3	H22(HD)	Asp302:OD2(OA)	1.938
					O13(OA)	Val338:HN(HD)	**1.780**
					H14(HD)	Val338:O(OA)	**1.992**
1	Eugenol dimer **14**	35	1-2	4	O25(OA)	Gln68:1HE2(HD)	2.248
					O13(OA)	Val338:HN(HD)	**2.105**
					O15(OA)	Val338:HN(HD)	**2.560**
					H14(HD)	Val338:O(OA)	**1.996**
2		19	1-2	3	O13(OA)	Ala303:HN(HD)	**2.483**
					O15(OA)	Ala303:HN(HD)	**2.130**
					H21(HD)	Val338:O(OA)	2.000
7		11	4	6	O13(OA)	Arg62:1HH2(HD)	**1.839**
					O15(OA)	Arg62:1HH1(HD)	**2.381**
					H21(HD)	Arg62:1HH2(HD)	**2.416**
					O13(OA)	Arg62:1HH1(HD)	**2.416**
					O15(OA)	Val98:O(OA)	2.171
					H21(HD)	Ser102:OG(OA)	1.774

2	Ferulic acid dimer **15**	8	1-2	3	O16(OA)	His308:HN(HD)	2.270
					O20(OA)	Val338:HN(HD)	**2.100**
					H21(HD)	Val338:O(OA)	**2.034**
4		25	1-2	4	O27(OA)	Gln68:1HE2(HD)	2.556
					H23(HD)	Tyr76:OH(OA)	2.568
					H23(HD)	Gly336:O(OA)	2.292
					H21(HD)	Val338:O(OA)	1.923
13		10	4-5	5	O16(OA)	Gln53:2HE2(HD)	2.386
					O18(OA)	Lys57:HZ2(HD)	**2.038**
					O18(OA)	Lys57:HZ3(HD)	**2.314**
					O20(OA)	Lys57:HZ3(HD)	**2.263**
					O27(OA)	Asp59:HN(HD)	2.181
4	**NPD352**	36	1-2	1	H41(HD)	Gln68:O(OA)	2.268

[a] Oxygen acceptor, [b] Hydrogen donor, [c] Cross-bridge H-bond interactions with the same aa are listed in bold.

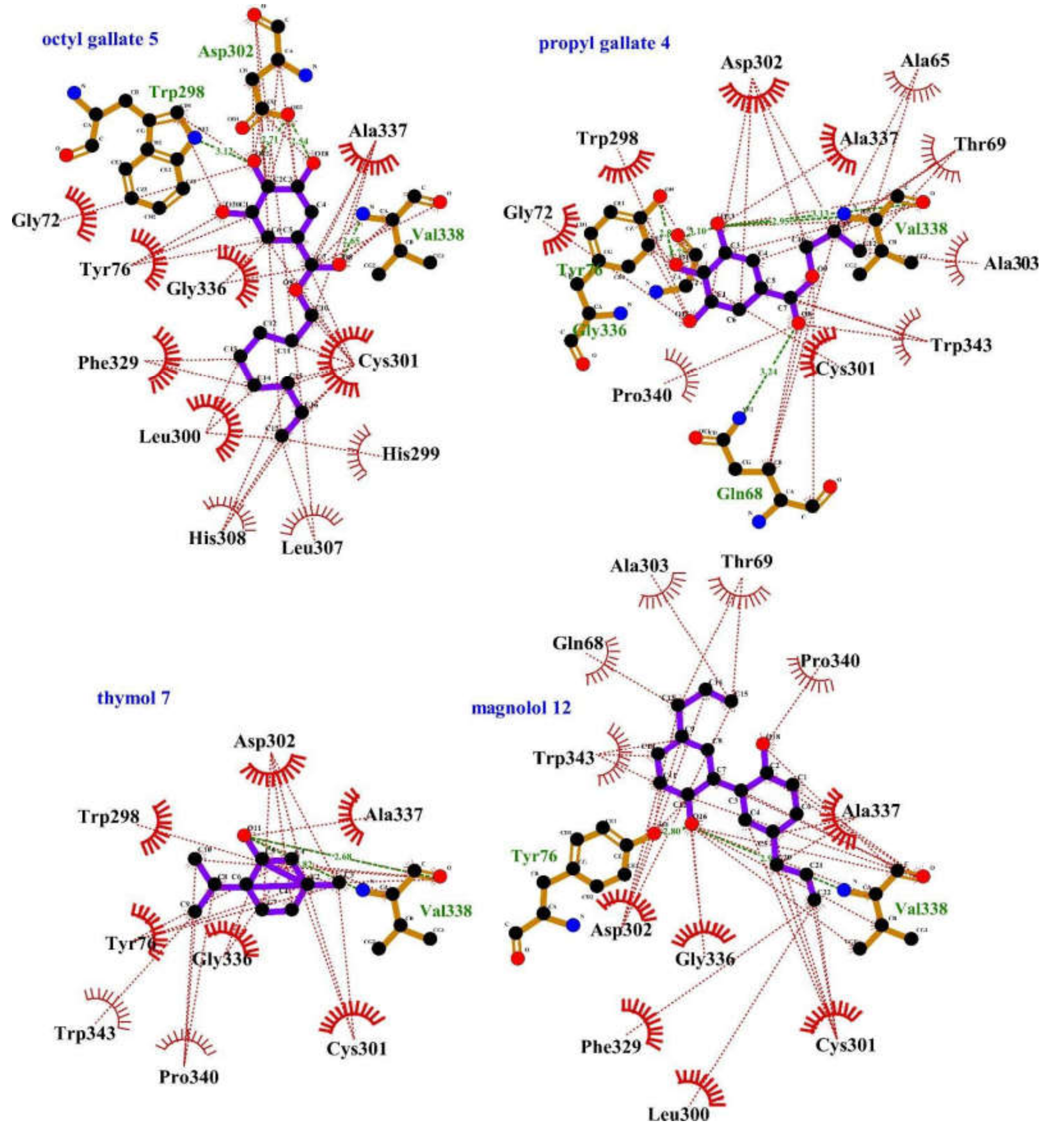

Figure S1. Estimated hydrophobic interactions of compounds **4**, **5**, **7** and **12** with the amino acids residues of TRI5-FPP.

Table S3. Estimated hydrophobic interactions of compounds **4**, **5**, **7** and 12 with the amino acids residues of TRI5-FPP illustrated in Figure S1.

			FPP		
**Tested Ligands**	**%**	**Sites**	**E.F.E.B. [a]**	**E.I.C., Ki [b]**	**Interactions**
Propyl gallate **4**	2	1	−5.72	64.62 uM	Gln68 Thr69 **Gly72** Tyr76 **Cys301** Asp302 **Gly336 Val338 Pro340 Trp343**
	39	1	−5.55	85.47 uM	**Ala65** Gln68 Thr69 Tyr76 **Cys301** Asp302 **Ala303 Gly336 Val338 Trp343**
	16	2	−5.29	131.61 uM	**Cys301** Asp302 **Ala303** Arg306 **Leu307** His308 **Phe329 Ala333 Ala337**
	7	1-2	−5.04	201.42 uM	**Gly72** Tyr76 **Cys301** Asp302 **Ala333 Gly336 Ala337 Val338**
	12	4	−4.36	632.98 uM	Lys57 **Val58** Asp59 Arg62 Ser103
Octyl gallate **5**	12	2-1	−6.53	16.47 uM	Tyr76 **Leu300 Cys301** Asp302 **Ala303** Tyr305 Arg306 **Leu307** His308 **Phe329 Ala333 Gly336 Ala337 Val338**
	15	1-2	−6.25	26.31 uM	**Gly72** Tyr76 **Leu300 Cys301** Asp302 Arg306 **Leu307** His308 **Phe329 Gly336 Ala337 Val338**
	10	1	−6.24	26.46 uM	**Ala65** Ser66 Gln68 Thr69 Tyr76 **Cys301** Asp302 **Ala303** Arg304 **Gly336 Val338 Trp343**
	9	2-1	−5.65	72.30 uM	Tyr76 **Leu300 Cys301** Asp302 **Ala303** Tyr305 Arg306 **Leu307** His308 **Phe329**
Thymol **7**	18	4	−5.51	91.27 uM	**Met55 Leu56** Lys57 **Val58 Val98 Leu99** Asp104 His125 **Pro126 Trp127**
	72	1	−5.34	120.99 uM	Tyr76 **Trp298 Cys301** Asp302 **Gly336 Val338 Pro340 Trp343**
	6	1	−5.14	171.69 uM	**Leu36 Gly72** Tyr76 **Cys301** Asp302 **Ala303 Gly336 Val338 Trp343**
Magnolol **12**	38	1-2	−6.93	8.36 uM	Gln68 Thr69 Tyr76 **Leu300 Cys301** Asp302 **Ala303 Phe329 Ala333 Gly336 Ala337 Val338 Pro340 Trp343**
	14	1-2	−6.79	10.59 uM	Gln68 Thr69 **Gly72** Tyr76 **Cys301** Asp302 **Ala303 Gly336 Ala337 Val338 Trp343**
	10	1-2	−6.46	18.40 uM	**Gly72** Tyr76 **Trp298 Leu300 Cys301** Asp302 His308 **Phe329 Gly336 Ala337 Val338 Pro340**
	17	4	−5.87	50.12 uM	**Met55 Leu56** Lys57 Arg62 **Val98** Ser102 Ser103 His125 **Pro126 Trp127**
Honokiol **13**	23	1-2	−7.25	4.89 uM	Gln68 Thr69 **Gly72 Trp298 Leu300 Cys301** Asp302 **Phe329 Ala337 Val338 Pro340 Trp343**
	13	2	−7.14	5.87 uM	**Leu300 Cys301** Asp302 **Ala303** Tyr305 Arg306 **Leu307** His308 Tyr311 **Phe329** Glu330 **Ala333 Ala337**
	23	1	−7.01	7.31 uM	**Leu36 Ala65** Gln68 Thr69 **Gly72** Tyr76 **Cys301** Asp302 **Ala303 Gly336 Ala337 Val338 Trp343**
	15	1-2	−6.93	8.36 uM	Gln68 Thr69 **Gly72** Tyr76 **Trp298 Cys301** Asp302 His308 **Phe329 Ala337 Val338**

[a] E.F.E.B.: Estimated Free Energy of Binding, [b] E.I.C., Ki: Estimated Inhibition Constant, Ki. Red and bold hydrophobic aa.

Table S4. Measurement of pH in liquid Vogel's medium amended with β-CD (3 mM) and different phenolic compounds at 0.25 mM after 0, 1, 3, 4, 5, 6, 8, 10 and 14 days (d) post inoculation with *F. culmorum* wild-type FcUK99 at 25 °C. *pKa of the carboxylic group; #pKa of the second phenolic-OH group.

**Treatment**	**Pka**	**Molarity (mM)**	**pH**								
			**0**	**1**	**3**	**4**	**5**	**6**	**8**	**10**	**14 days**
Control	--	--	5.74	5.77	6.79	6.76	6.68	6.99	7.41	7.98	8.17
p-Coumaric acid **1**	4.65*	0.25	5.70	5.79	6.77	7.00	7.09	7.08	7.12	7.37	6.62
Caffeic acid **2**	4.80*	0.25	5.69	5.68	6.61	6.64	6.75	7.05	7.46	7.96	8.10
Ferulic acid **3**	4.61*	0.25	5.68	5.70	6.64	6.85	6.94	6.97	6.85	6.93	7.23
Propyl gallate **4**	7.94	0.25	5.76	5.74	5.53	5.32	4.90	4.77	5.05	5.25	5.89
Octyl gallate **5**	8.11	0.25	5.73	5.81	6.90	7.14	7.22	7.22	7.21	7.40	6.55
Carvacrol **6**	10.42	0.25	5.74	5.72	6.8	6.77	6.90	7.27	7.57	8.08	8.25
Thymol **7**	10.59	0.25	5.83	5.89	5.59	5.33	5.02	5.01	4.87	5.28	6.02
Linalool **8**	18.45	0.25	5.70	5.75	6.8	6.97	7.04	6.94	6.97	7.56	8.02
Geraniol **9**	14.45	0.25	5.71	5.72	6.71	6.97	7.36	7.49	7.55	7.96	8.17
Apocynin **10**	8.17	0.25	5.73	5.79	6.34	6.50	6.66	6.71	6.94	7.36	6.82
Eugenol **11**	10.19	0.25	5.76	5.71	6.84	6.94	6.89	6.9	7.38	7.94	8.19
Magnolol **12**	7.10, 10.58#	0.25	5.74	5.84	7.03	7.17	7.24	7.18	7.18	7.36	6.76
Honokiol **13**	9.64, 10.71#	0.25	5.71	5.80	6.31	6.45	6.63	6.66	6.89	7.31	6.70

Eugenol dimer **14**	N.D.	0.25	5.73	5.75	6.90	6.79	6.90	7.13	7.55	8.05	8.20
Ferulic acid dimer **15**	N.D.	0.25	5.65	5.69	6.65	7.00	7.35	7.43	7.71	8.15	8.40
Propyl gallate **4** +thymol **7**	--	0.125 + 0.125	5.85	5.93	5.75	5.67	5.39	5.33	5.31	6.03	6.90
Magnolol **12** + thymol **7**	--	0.125 + 0.125	5.72	5.79	7.01	7.12	7.23	7.18	7.16	7.38	6.72
Honokiol **13**+ thymol **7**	--	0.125 + 0.125	5.74	5.77	6.30	6.51	6.70	6.89	7.21	7.41	6.81
